# The challenge of mothers learning about secondhand smoke (MLASS): a quasi-experimental, mixed methods feasibility study

**DOI:** 10.1186/s40814-016-0048-0

**Published:** 2016-02-06

**Authors:** Rachel Mann, Heather Thomson, Becky Reynolds, Amanda Amos, Kamran Siddiqi

**Affiliations:** 1 Social Policy Research Unit, Alcuin College, University of York, Heslington, York, YO10 5DD UK; 2 The Office of the Director of Public Health, Technorth, 9 Harrogate Road, Chapel Allerton, Leeds, LS7 3NB UK; 3 Public Heath Team, Bath & North East Somerset Council, St Martin’s Hospital, Clara Cross Lane, Bath, BA2 5RP UK; 4 Usher Institute of Population Health Sciences and Informatics, Medical School, University of Edinburgh, Teviot Place, Edinburgh, EH8 9AG UK; 5 Department of Health Sciences, University of York, ARRC Building, 1st Floor, Heslington, York, YO10 5DD UK

## Abstract

**Background:**

Exposure to secondhand smoke (SHS) in the antenatal and postnatal period is associated with a detrimental health impact to the foetus and newborn baby and is recognised as a preventable public health challenge. The aim of the mother’s learning about secondhand smoke (MLASS) study was to test the feasibility of delivering and evaluating the effectiveness of a smoke-free homes (SFH) health education intervention in the antenatal and postnatal period to reduce foetal and newborn exposure to SHS.

**Methods:**

Pregnant women aged 17–40 years old who attended their first community-based antenatal appointment in Leeds, UK, were eligible to participate if they currently smoked, or if they were non-smokers but lived in a household where someone else smoked, or had regular visitors to the home who smoked. A SFH health education intervention was delivered at four time points by community midwives and health visitors. Outcome measures included self-reported level of household smoking restrictions and SHS exposure in pregnant women who did not smoke during pregnancy and in the newborn baby, measured by salivary and urine cotinine levels, respectively. We planned to conduct focus group discussions with participants and health professionals. A post hoc survey of pregnant women was conducted at the recruitment site.

**Results:**

Eight pregnant women were recruited over a 6-month recruitment period. Of the 65 eligible pregnant women approached, 57 (88 %) declined to participate in the study. The majority declined to participate due to lack of interest in the study. In the post hoc survey, the majority of pregnant women reported that they were already implementing household smoking restrictions to reduce SHS; only a small number had no household smoking restrictions.

**Conclusions:**

The post hoc survey identified women who could benefit from a SFH intervention; therefore, future studies should consider what SFH means to pregnant women and may wish to target those not currently implementing household smoking restrictions. Future recruitment strategies in studies of an SFH intervention in the context of maternity service pressures needs careful consideration; this includes the capacity to undertake the research, the recruitment setting, the criteria for individuals requiring the intervention, and individuals’ willingness to engage with such research.

**Electronic supplementary material:**

The online version of this article (doi:10.1186/s40814-016-0048-0) contains supplementary material, which is available to authorized users.

## Background

Smoking in pregnancy is a leading preventable cause of foetal, obstetric and neonatal morbidity and mortality associated with numerous adverse outcomes including poor foetal intra-uterine growth and neuro-development, placental abruption, miscarriage, preterm birth and low birth weight [1]. Likewise, exposure to secondhand smoke (SHS) in the antenatal and in postnatal period is associated with a detrimental impact to the foetus and newborn baby and is recognised as a preventable public health challenge [2].

SHS is the inhalation of other people’s tobacco smoke commonly known as ‘passive smoking’; other terms include ‘environmental tobacco smoke’ and ‘involuntary smoking’ [3]. There are two types of SHS, ‘side stream’ smoke from the burning tip of a cigarette and exhaled ‘mainstream’ smoke exhaled by the smoker [4]. Toxins inhaled in both mainstream and side stream smoke are substantial. Over 4000 chemicals (both particles and gases) including chemical irritants and almost 70 carcinogens are inhaled in mainstream smoke by smokers [5]. Whilst side stream has a similar composition to mainstream smoke, the concentrations of toxins and carcinogens in side stream smoke have been found to be substantially higher [6].

Prevalence of self-reported SHS exposure during pregnancy in a large UK cohort study has been estimated as 13 % [7] and in low- and middle-income countries between 9.3 and 82.9 % [8]. In the UK, approximately 50 % of all newborns are exposed to tobacco smoke due to maternal smoking or contact with SHS [7]. Globally, an estimated 700 million children, almost half of the world’s child population, are thought to be exposed to SHS [9].

Women’s exposure to SHS during pregnancy reduces infants’ adjusted mean birth weights by on average 36 g and increases the risks of babies being small for gestational age or low birth weight at term [7]. Newborns and infants exposed to SHS after birth are also at increased risk of acute lower respiratory infections, middle ear infections, SIDS, meningococcal disease, developing and exacerbating asthma, increased frequency of hospital visits, persisting wheeze and reduced lung function [10].

Although legislation to ban smoking in enclosed public places and workplaces has been adopted in several countries, including the UK since 2007 [11–13], the majority of SHS exposure experienced by pregnant women and children including newborns occurs at home [13, 14].

Pregnancy and parenthood has been identified as a life event that can influence health-related beliefs, attitudes and behaviours, particularly as early pregnancy and parenthood offer the opportunity for health education interventions when there is heightened awareness of health risks to the pregnant woman, unborn foetus and newborn baby [15]. Recognition of the potential risks to a child’s health has been identified as a major determinant of families agreeing to implement smoking restrictions [16]. It has been suggested that developing parents’ confidence in providing a smoke free environment and offering to support them in achieving this goal is likely to be effective [17].

However, the evidence to support specific measures to implement smoking restrictions and reduce SHS exposure at home in the antenatal and neonatal period is limited due to lack of research using objective outcomes measurements, theory-driven interventions and appropriate settings to offer SHS-related advice [18, 19].

A recent systematic review evaluated five randomised controlled trials (RCT) that compared usual care with psychosocial interventions to assess reduction in SHS exposure in non-smoking pregnant women, including one also offering cessation support (pharmacological) for smoking partners [20]. However, the poor study quality of three trials due to lack of biochemical validation using cotinine levels of self-reported exposure to SHS limit recommendations regarding the effectiveness of one intervention over another.

An additional limitation of studies that have evaluated strategies to reduce SHS exposure in antenatal and postnatal periods that have reported objective biochemical outcome measures is that only a very small minority assess both maternal and infant cotinine levels to evaluate SHS exposure.

In the UK, promoting smoke-free homes is a national priority as highlighted in several policy documents including ‘Beyond Smoking Kills’ [21], ‘Passive Smoking and Children’ [22] and NICE guidance on smoking and pregnancy [23]. More recently, a report by the World Health Organisation strongly recommended that routine screening should be undertaken for SHS exposure in pregnant women attending routine antenatal care, and interventions should be targeted at pregnant women to reduce the exposure to SHS in pregnancy [24].

We developed a smoke-free homes (SFH) intervention in consultation with pregnant women, new mothers and health professionals designed to help pregnant women and new mothers learn about the hazards of SHS, evaluate their own smoking behaviour and empower them to negotiate smoking restrictions at home. The mother’s learning about secondhand smoke (MLASS) study aim was to test the feasibility of delivering and evaluating the effectiveness of a SFH health education intervention with (i) non-smoking pregnant women to reduce their exposure to SHS in the home and reduce foetal exposure to SHS and (ii) with new mothers (irrespective of their smoking status) to reduce newborn babies exposure to SHS in the home. In addition, we conducted a brief post hoc survey in another group of pregnant women to identify the extent to which they received smoke-free homes information from their community midwife and implement smoking restrictions in their homes.

## Methods

We planned to test out the feasibility of the intervention and its evaluation by piloting it with women and their newborns using a quasi-experimental mixed methods approach consisting of two elements, a quantitative before-and-after study and a qualitative study in order to (i) test its fidelity, appropriateness and acceptability, (ii) investigate the key constraints and drivers in delivering a SFH intervention and (iii) optimise parameters (e.g. recruitment, outcomes measurements) to strengthen the design of a future trial.

**Fig. 1:**
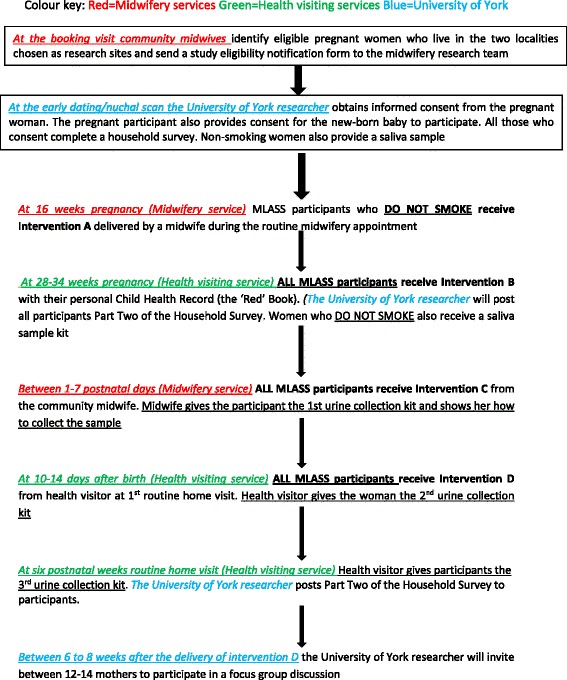
MLASS study pathway

Figure 1 displays a summary of the study pathway, and this includes details of participant recruitment and the time points for data collection and delivery of the SFH intervention.

### Quantitative study

#### Participants and setting

Pregnant women aged 17–40 years old were eligible to participate if (i) they currently smoked or (ii) if they were non-smokers who lived in a household where someone else smoked (e.g. partner, parent or other family member) or had regular visitors to the home who smoked and (iii) they resided among two deprived localities with a higher than average prevalence of household smoking in Leeds, UK. Women were excluded from the study if they did not smoke and did not live in a smoking household. No other study exclusion criteria were imposed.

In the UK, most maternity care is provided by the National Health Service (NHS) where 96.7 % of women access and book their maternity care via their general practitioner or community midwife team [25]. In the NHS, the majority of antenatal care is provided by community midwives and postnatal care provided by community midwives for the first 10 days after delivery and then by community health visitors. The two localities chosen as the research sites are representative of the NHS maternity services landscape as they are served by community midwifery teams that deliver antenatal maternity care at community health centres (CHCs) and provide postnatal maternity care between 1 and 7 days postdelivery in both the home and CHCs, and health visiting teams delivering child and family health services in the home and at CHCs from 28 weeks pregnancy through to the child’s fifth year.

#### Intervention

In the phase 1 developmental stage of the project, development of the intervention was based on behaviour change techniques included in a 26-item taxonomy [26] developed from behaviour change theories such as Social Cognitive Theory, Theory of Planned Behaviour and Theory of Reasoned Action. According to Abraham and Michie (2007) [26], the taxonomy provides a pre-defined set of distinct, theory-linked definitions of behaviour change techniques (BCTs) so theories that specify the same process of behaviour change imply the same BCT, and therefore, use of the taxonomy supplements intervention description by providing a list of BCTs and the accompanying theoretical underpinning and not just a description of the mode of intervention delivery and type of person delivering the intervention component. The taxonomy provides a theoretical framework to describe the intervention components, and Abraham and Michie acknowledge that further work is required to translate theories relevant to behaviour change into specific change techniques [26]. The taxonomy has since been updated [27].

In the phase 1 stage, two of the authors (HT and BR) mapped proposed intervention components to the abovementioned 26-item taxonomy [26]. Additional file 1 displays the mapping exercise and demonstrates which of the BCTs were mapped to proposed potential activities and techniques in order to describe each component included in the intervention, as recommended by Abraham and Michie [26].

The intervention was further developed during consultation with over 100 pregnant women and new mothers attending child health centres in Leeds, UK, and 16 health professionals from midwifery and health visiting services between September 2011 and January 2012. Focus group discussions were conducted with women to elicit current knowledge about the hazards of SHS and their views about the content and delivery of the intervention. Separate focus groups were conducted with health professionals regarding the content and delivery of the intervention. Emergent themes from focus groups with women were investigated in a semi-structured questionnaire; health professionals also completed a version of this questionnaire. Details of focus group discussion topic guides and questionnaires are presented in Additional file 2.

The key elements that emerged from the consultations with the new mothers and health professionals identified that the intervention needed to be delivered in routine care and at different time points during the antenatal and postnatal period, with both midwives and health visitors involved in the delivery and that women required support on how to influence smokers in the family to reduce newborn exposure to SHS and also factual information about the harms and consequences of SHS exposure.

The phase 1 consultation process resulted in the creation of a health education intervention to be delivered by community midwifery services and health visitor services at three or four time points (dependent on smoking status) during pregnancy and in the first ten postnatal days after delivery.

The study intervention comprised:

- *Intervention A*: a heat-sensitive (images only appear on touch), educational leaflet which can be used in interactive sessions to be inserted into the pregnant woman’s maternity notes. This intervention is focused on supporting the woman to avoid SHS during pregnancy in order to protect the developing foetus. Activating the heat-sensitive component was designed to reveal a hidden message to ‘imitate’ the action of clearing away a cloud of smoke and provoke a cognitive reaction among pregnant women to act and protect their foetus from secondhand smoke.
- *Intervention B*: an educational leaflet focused on raising awareness of the harms of secondhand smoke and ways to protect the newborn baby from secondhand smoke after birth.
- *Intervention C*: a magnetic concertina credit card-sized piece of print that the woman can give away to family and friends to ask them not to smoke around the newborn baby.
- *Intervention D*: an educational booklet written from the perspective of the baby telling a story of welcoming the baby into the home. The intervention is aimed at supporting the new mother in reinforcing smoke-free homes messages.

A copy of each intervention is displayed in Additional file 3.

Women received SFH information from their community midwife and health visitor at routine antenatal and postnatal appointments. SFH information was delivered at four time points between 16 weeks of pregnancy and 10 days after the baby was born. Community midwives were responsible for delivering interventions A and C, and health visitors were responsible for delivering interventions B and D. The intervention delivery involved the health professional giving the woman the educational resource and a brief conversation signposting her to the information it contained. Timing of the intervention delivery and the service responsible for delivery of each intervention was based on advice from the midwifery and health visiting services that agreed to host the research sites. Timings of intervention delivery and choice of service were based on each service identifying key maternity care contact points during routine care with women across the antenatal and postnatal care pathway where it would be feasible to deliver the educational resources in routine practice. The key contact points identified were at:

- 16 weeks pregnancy; all women attend an appointment with their community midwife after confirmation of their pregnancy via the first ultrasound scan. This appointment was therefore the earliest opportunity to deliver the first intervention (intervention A) to protect the pregnant mother from SHS exposure and the unborn foetus.
- 28 weeks pregnancy; all women receive a routine ‘Early start’ contact with health visiting services. The routine contact is designed to introduce the new mother to the service and discuss the mother and future baby’s well-being and the overall family health and circumstances; therefore, this key contact was ideal to deliver the second intervention focused on protecting the newborn baby when born from SHS exposure.
- third postnatal day; when all women receive a routine visit from their community midwife and this represented the earliest postnatal opportunity to deliver a third intervention (intervention C) to remind the new mother about the harms of SHS exposure to the newborn baby.
- tenth postnatal day; when all women receive a routine home visit from their health visitor and this contact represented the next earliest opportunity in routine practice to deliver a fourth intervention (intervention D) to re-inforce the smoke-free homes message in the early postnatal period.

#### Measures

The primary outcome measure was exposure to SHS. This was measured in two ways:

- Antenatal primary outcome measure
  The primary antenatal outcome was measured in pregnant women who did not smoke during pregnancy (but living with a partner who smokes and/or have regular visitors to the house who smoke) was salivary cotinine levels. Saliva samples were collected at study entry (baseline) and at 28–32 weeks gestation. Saliva samples were collected using a Sarstedt Salivette (www.sarstedt.com). Non-smoking pregnant women were asked to place a small dental roll in their mouth for approximately 3 to 5 min until saturated, which was then placed in the salivette container. Baseline saliva samples were collected in the antenatal clinic with the help of the researcher. Follow-up sample packs were mailed to participants, who collected the sample and mailed it directly to the laboratory. Measurement of secondhand smoke exposure in those women who smoked in pregnancy was not undertaken—any measurement of SHS exposure would be irrelevant as their cotinine levels would confirm they smoked.
- Postnatal outcome measure
  The primary postnatal outcome, which was measured in newborn babies, irrespective of their mother’s smoking status, was urine cotinine levels at three postnatal time points. Postnatal time points specified for urine sample collection were between 1–7 postnatal days, 10–14 postnatal days and 6–8 weeks after baby’s delivery. Urine samples from newborns were collected by the new mother at home by use of a urine collection kit provided by the research team and delivered by the midwives and health visitors at the time the intervention was delivered. The method of urine collection comprised cotton wool balls placed in the baby’s nappy (diaper) close to the urethra. After the baby passed urine, the urine-soaked cotton wool balls were placed inside a 50-ml plastic syringe and the urine was expressed into a small container. Mothers were asked to only collect urine samples from ‘clean’ nappies not contaminated with baby’s faeces. A printed instruction leaflet for the collection of urine samples was included in each urine collection kit. Urine samples were posted directly to the laboratory in a container approved for mailing biological samples.

The saliva and urine samples were analysed by gas-liquid chromatography technique that can detect cotinine levels as low as 0.1 ng/ml [28]. Cotinine levels detected in non-smoking pregnant women and newborns indicate SHS exposure. The interpretation of salivary and urine cotinine levels was based on defined cut points: interpretation of pregnant women’s salivary cotinine levels (ng/ml) <0.1 = no exposure to SHS, 0.1–12 = exposure to SHS and >12 = smoker [29]. The interpretation of newborn babies urine cotinine levels (ng/ml) are as follows: <0.1 = no exposure to SHS, 0.1–50 = exposure to SHS, and >50 = smoker [30].

Demographic data were collected at baseline, and the level of smoking restrictions at home was measured through a self-report survey completed by the women at baseline, 28–32 weeks of pregnancy and between 6 and 8 postnatal weeks after delivery.

#### Sample size

We planned to recruit a purposive sample of 200 pregnant women and their unborn babies. A formal sample size calculation was not performed; we planned to recruit 200 pregnant women in order to explore the potential target population and assist with decisions regarding future research work around the target population and intervention delivery parameters.

#### Recruitment and follow-up procedure

Recruitment was conducted over a 6-month period from September 2013 to February 2014. Women were identified as eligible to participate by a community midwife (CMW) at the time they attended their first midwifery appointment in a community health centre to confirm their pregnancy (gestation approximately 6 weeks) and book their maternity care (the ‘booking visit’). This includes a referral for the first routine ultrasound scan (USS) undertaken at approximately 12 weeks gestation to establish the estimated date of delivery (EDD). The first USS takes place at either of the two general hospitals that provide maternity services to the residents of Leeds, UK, within the Leeds Teaching Hospitals National Health Service Trust (LTHT). As part of the booking visit in LTHT, CMWs routinely ask questions about pregnant women’s and their partners’ smoking status. Pregnant women who were eligible to participate were informed by the CMW about the study, given a study information pack and informed that a researcher would approach them in the antenatal clinic within the hospital after their first routine ultrasound scan to establish the EDD. A study referral form for eligible women was sent by the CMW to the research midwifery team located at the two general hospitals, in order to identify the date and time of the USS for each potential participant and inform the researcher to attend the appointment. After the USS appointment, the MLASS researcher (RM) approached the potential participant and enquired if they wished to participate in the study. For those pregnant women who agreed to participate written informed consent was obtained by the researcher (RM); baseline data (demographic data, self-reported household smoking restrictions and salivary cotinine of non-smoking pregnant women) was obtained. At study entry, participants also provided written informed consent on behalf of their unborn babies. Pregnant women who declined to participate were invited to complete an anonymous decline form to indicate their reasons for non-participation. Eligible pregnant women who declined to participate were asked to endorse as many reasons as they wished from a pre-defined list of decline statements. Women who declined could also complete free text comments about their reason for decline. An additional opportunistic recruitment strategy was also implemented, whereby all dating scan bookings at the antenatal clinics for the two study sites were notified to the researcher (RM) via additional searching of scan bookings via the NHS booking systems at the recruitment site by the research midwife team at LTHT.

Ethical approval for the study was granted by Yorkshire and the Humber Research Ethics Committee (Reference: 12/YH/0257).

#### Analysis

The descriptive characteristics of the study sample using proportions where required were used to analyse these data and, if appropriate, bivariate analysis of key variables were conducted.

### Qualitative study

A post-intervention qualitative study was undertaken with the health professionals who delivered the intervention and the women who received it to elicit their views on the appropriateness, acceptability and feasibility of the intervention in this setting.

We planned to invite women to a focus group discussion (FGD) post-intervention. We intended to invite a purposive sub-sample of 12–14 women based on the following: (i) socio-demographic characteristics, (ii) women who received the intervention antenatally, (iii) women who did not receive the intervention antenatally, (iv) women from households that implemented changes in the home to protect themselves whilst pregnant/protect the newborn, and (v) women from households that did not implement changes in the home to protect themselves whilst pregnant/protect the newborn. Topics included understanding to what extent mothers felt supported through the smoke-free homes intervention, which factors motivated them, and which were their main constraints.

We planned to invite CMW and HV health professionals who had delivered the intervention to a separate FGD. Topics were designed to gain an in-depth understanding of the way the intervention was implemented, which factors acted as barriers to effective implementation and how health professionals overcame these.

We planned to record qualitative interviews using a digital recorder, transcribe interview verbatim, then code data and analyse these data using thematic analysis. [31].

### Post hoc survey method

An anonymous self-report survey was conducted after MLASS data collection ended with pregnant women who attended for US scan between April and June 2014 at the LTHT antenatal clinic in order to identify to what extent pregnant women (a) already received information about smoke-free homes from their CMW and (b) implement smoking restrictions in the home (see Additional file 4). Pregnant women were invited by the researcher (RM) to complete the brief anonymous self-report survey. All pregnant women who attended for US scan were eligible to complete the survey.

## Results

Pregnant women were recruited over a 6-month period between October 2013 and February 2014. Table 1 displays the number of pregnant women identified, approached and recruited from the different recruitment strategies. Over the 6-month recruitment period, only eight pregnant women consented to participate in the study. In the first 10 weeks of the recruitment period, only six study eligibility referrals were sent by the community midwives to the research team (see Table 1).

**Table 1:** Study recruitment

	Number of pregnant women
Total number of study eligibility notification forms sent by community midwives during 6-month recruitment period	54
Additional USS bookings identified by antenatal administration team	178
Total number of pregnant women identified from research sites during 6-month recruitment period	232
Total number of women approached in antenatal clinic	131^a^
Number of pregnant women eligible to participate	65^b^
Number of smokers approached	41
Number of eligible non-smokers approached	24
Number of eligible pregnant women declined	57
Number of eligible pregnant women consented	8

^a^101 pregnant women were not approached by the researcher (63 = researcher unable to attend; 36 = other reasons, e.g. failed pregnancy; patient did not attend)

^b^Non-eligible pregnant women approached = 66 (63 = non-smokers, 2 = failed pregnancy, 1 = moved from study site area)

Of the 65 eligible women approached, a total of 57 (88 %) pregnant women declined to participate in the study.

Forty-nine decline forms were received (86 % response rate) comprising a total of 114 reasons for declining (see Table 2).

**Table 2:** Eligible pregnant women’s reasons for decline

Reason for decline	Number of responses
I am not interested in taking part in this study	33
I do not want to receive educational information about smoke free home	9
I do not think smoke free homes is an important issue	3
I do not have time to take part in this study	26
I do not feel well enough to take part in this study	4
I think it would be too much commitment to take part in this study	20
Other reasons:	
Don’t want to give/collect baby urine samples	5
We already have a smoke free home	7
Other reason (*n* = 3)/family commitments (*n* = 4)	7

An additional question on the decline form asked eligible pregnant women if they would be more likely to participate if they were offered a financial incentive. The following responses were received: ‘No’ = 42 women, ‘Yes’ = 3 women, and ‘Not sure’ = 4 women.

All eight participants, who consented to participate in the MLASS study, completed the self-report survey at study entry, which collected demographic data and household smoking information. The age range of participants at study entry (baseline) was 17–30 years of age; five participants were ≤21 years old. Four participants reported that they were current smokers and four were non-smokers who lived with a partner who smoked or another family member (i.e. parent) who smoked. All eight participants reported their ethnicity as white. Two participants were in full-time employment, two were unemployed and four were students. Level of education reported by participants comprised left school at 16 with no qualifications = 1, left school at 16 with some qualifications = 2, left school at 18 with some qualifications = 4 and higher education (e.g. Bachelor’s degree) = 1.

Table 3 displays the outcomes data the study was able to collect from the eight participants.

**Table 3:** Participant outcomes data collection

Participant smoking status	Saliva samples received	30-week pregnancy F/up survey received	Baby’s urine samples received after delivery	6–8-week postnatal F/up (survey and interview)
Smoker	N/A	No	One sample received 23 PN weeks after delivery	Lost to F/up
Non-smoker	Baseline only	No	None	Lost to F/up
Smoker	N/A	Yes	One sample received dated PN day 14	No survey. Interview completed.
Smoker	N/A	No	None	Lost to F/up
Non-smoker	Baseline only	No	None	Lost to F/up
Non-smoker	Baseline and at 30 weeks gestation	Yes	None	Survey completed. Declined to participate in interview.
Non-smoker	Baseline and at 30 weeks gestation	Yes	Sample 1 received dated PN day 6 Sample 2 received dated PN day 12 Sample 3 received dated 7 PN weeks	Survey completed. Interview completed.
Smoker	N/A	Yes	One sample received 3 PN weeks after delivery	Lost to F/up

*F/up* follow-up, *PN* postnatal

A total of seven participants provided either saliva samples and/or baby’s urine samples for analysis to determine the presence or absence of cotinine and exposure to SHS. Table 4 displays the results from the participants who provided samples and an interpretation of these data. A total of four participants provided samples of their baby’s urine; however, only one participant collected three urine samples from her baby as per the study protocol. The table shows that all four infants were exposed to SHS.

**Table 4:** Results of salivary samples and urine samples of study participants

Participant smoking status	Saliva samples received from pregnant women	Saliva cotinine result (ng/ml)	Saliva: level of exposure to secondhand smoke (SHS)	Baby’s urine samples received after delivery	Urine cotinine result (ng/ml)	Urine: level of exposure to secondhand smoke (SHS)
Smoker	N/A	N/A	–	One sample received 23 PN weeks after delivery	20.9	Exposure to SHS
Non-smoker	Baseline only	155.3	Smoker	None	–	–
Smoker	N/A	N/A	–	One sample received dated PN day 14	5.5	Exposure to SHS
Non-smoker	Baseline only	1.1	Exposure to SHS	None	–	–
Non-smoker	Sample 1 at baseline	0.2	No exposure to SHS	None	–	–
	Sample 2 at F/up	<0.1	No exposure to SHS			
Non-smoker	Sample 1 at baseline	<0.1	No exposure to SHS	Urine sample 1 received dated PN day 6	<0.1	No exposure to SHS
	Sample 2 at F/up	<0.1	No exposure to SHS	Urine sample 2 received dated PN day 12	<0.1	No exposure to SHS
				Urine sample 3 received dated at 7 PN weeks	1.3	Exposure to SHS
Smoker	N/A	N/A	–	One sample received 3 PN weeks after delivery	10.3	Exposure to SHS

*F/up* follow-up, *PN* postnatal

There were no adverse outcomes, such as miscarriage, stillbirth or serious hospitalization during the MLASS study associated with the participant’s pregnancy, at the time of the baby’s birth or in the 8-week study period after the baby’s birth. All eight participants delivered their babies between March and August 2014. Adherence by health professionals to the intervention delivery schedule is displayed in Additional file 5.

### Qualitative study

We intended to conduct an FGD with participants and a separate FGD with health professionals to find out their views on the appropriateness, acceptability and feasibility of the SFH intervention in this setting. Poor overall recruitment meant that an FGD with participants was not feasible. We sought to undertake face-to-face interviews; however, only two participants agreed to interview. A FGD with health professionals was not possible due to NHS service constraints (reasons included time and case-load pressures); however, we were able to conduct a face-to-face interview with one health professional from each participating service.

Due to the limited data available from the small number of face-to-face interviews that were conducted, we cannot draw any transferable conclusions as to the usefulness and acceptability of the SFH advice to a wider population of women in other antenatal and postnatal settings or indeed the capacity of other maternity services and health visiting services to deliver a SFH intervention in other maternal and child healthcare service settings.

### Post hoc survey results

Given that participant recruitment was so challenging and only eight pregnant women consented to participate, a post hoc survey among another group of pregnant women, after the MLASS study data collection period, helped us to contextualise our findings.

All 542 pregnant women approached by the researcher (RM) completed the survey. Pregnant women who completed the survey were 9–39 weeks gestation (average gestation = 23 weeks); 68 % (368) pregnant women reported that they did not currently smoke or lived with a smoker, and 17 % (91) pregnant women reported that they currently smoked. There were a total of 32 % (174) smoking households comprised of the following: pregnant women who smoke living with a non-smoking partner = 14, pregnant women who smoke living with a smoker/regular visitors of house who smoke = 77 and non-smoking pregnant women who live with a partner who smokes/regular visitors to the house who smoke = 83.

From the 174 smoking households, 80 % (139) pregnant women reported that they had received information related to SHS from their CMW, 2 % (3) reported that information was received from another source and 18 % (32) reported that they had not received any such information. Of the 139 pregnant women who reported receiving smoke-free homes information from their CMW, 91 % recalled either receiving the smoking cessation information that forms part of their handheld maternity notes at the booking visit, receiving NHS smoking cessation leaflets or receiving verbal advice from their CMW to refrain from smoking inside the house. Pregnant women reported the following information on smoking restrictions implemented in their household (see Table 5).

**Table 5:** Household smoking restrictions

Response choices	Number of responses (*n* = 174)
My home was smoke free even before I found out I was pregnant	85 (49 %)
My home has been smoke free since I found out I was pregnant	55 (32 %)
There are no smoking restrictions in my house	31 (18 %)
Additional comments provided: Smoking is restricted to the kitchen	3 (1.7 %)

## Discussion

The MLASS study sought to examine the feasibility of delivering a SFH intervention in antenatal and in postnatal period and to examine key uncertainties such as recruitment and retention, fidelity of the intervention, appropriateness of outcomes and validity of the research tools. We found the study extremely challenging due to difficulties encountered in recruiting participants, primarily due to the lack of interest of eligible pregnant women in the study. Retention of participants within the study and collection of outcomes data such as saliva and urine samples was also difficult.

This feasibility study design included both (i) smoking and non-smoking pregnant women and (ii) community midwives (MWs) and health visitors (HVs) for intervention delivery, and (iii) the study was designed to test the feasibility of delivering an intervention across the antenatal and early postnatal pathway. At the time of designing the study, it was not known how many women would be the optimum number to recruit; therefore, a precise sample size was not estimated. We did not know in which part of the maternal care pathway would it be the optimum to deliver the intervention, whether indeed the intervention could be delivered by MWs and HVs and also whether one particular participant group might be more interested in participating or need/request additional support to implement smoking restrictions in their home, for example non-smoking pregnant women. We believed that if we aimed to recruit a sample of 200 pregnant women, we could explore the potential target population and that a sample of this size would allow us to make a definitive decision as to how to proceed in terms of our target population and intervention delivery parameters if the feasibility study moved forward to a pilot cluster randomised controlled trial.

Challenges encountered in the study recruitment demonstrated that in the context of the local community midwifery service, it was not feasible for CMWs to identify sufficient numbers of eligible pregnant to achieve a study sample size of 200 pregnant women in the course of routine community antenatal care appointments.

It was particularly difficult to engage with all of the potential CMWs to participate in the identification of eligible pregnant women in the context of their routine care of pregnant women, and in the first 10 weeks of recruitment, only six eligibility referrals were sent to the research team. There were fewer numbers of referrals of eligible pregnant women received than expected from the CMWs; therefore, there was a much smaller pool of pregnant women available for the researcher to approach for recruitment. Discussions with CMWs about the reasons for lack of eligibility referrals found that the constraints and time pressures at the booking visit, which is the first antenatal care appointment that women have with their community midwife, meant that it was difficult to add an additional task, there was lack of motivation among pregnant women to engage in a study about SHS, many of the pregnant women approached by CMWs questioned whether there was any benefit of taking part in the study and many pregnant women refused to take the study literature when offered.

There were also issues with the opportunistic recruitment strategy. The researcher was unable to ascertain smoking status prior to the USS appointment, and therefore, a significant amount of time was wasted approaching women who did not smoke and did not live with a smoker; this accounted for a total of 66 women of the 131 women approached who were not eligible to participate due to their non-smoking status. In addition, the opportunistic strategy meant that pregnant women were approached with no prior warning of the study, so prior to researcher’s approach, women had no opportunity to consider the research, which may have affected pregnant women’s willingness to consider study participation. In addition, our local comprehensive local research network did not have any funds available to facilitate recruitment in MLASS study and despite it being accepted as a National Institute for Health Research (NIHR) portfolio study—a status that usually confers assistance with study recruitment and study activities.

The other main factor associated with poor recruitment was the lack of pregnant women’s willingness to engage with the study; only eight pregnant women consented to participate. The majority of eligible women who were approached reported that they were not interested in participating, and the two other most common reasons for declining were cited as lack of time and too much commitment expected in the study. Women who declined to participate indicated that they would be unlikely to participate in the study even if they were offered a financial incentive. Of the eight women who consented to participate, five were lost to study follow-up in the postnatal period with regard to the follow-up survey and the face-to-face interview. This is despite antenatal non-responders receiving three non-response reminders as necessary, which included one text message reminder, a follow-up survey and covering letter and a reminder telephone call and postnatal non-responders who received two text message reminders.

Adherence to the delivery of the SFH intervention by health professionals was monitored (Additional file 5). Three out of the four non-smoking pregnant women received intervention A as per protocol at approximately 16 weeks of pregnancy. There was excellent adherence to the delivery of interventions B and D by health visitors; all interventions given by health visitors were delivered as per specified protocol time points. However, there were difficulties with adherence to the delivery of intervention C by CMWs between day 1 and day 7 after the baby’s birth. Only one participant received the intervention as per protocol, and therefore, it is difficult to draw any conclusions as to the capacity of the midwifery service to deliver this information. Challenges cited by CMWs that were identified in poor adherence to the delivery of intervention C included service constraints such as time pressure, large caseloads, not all new mothers are routinely seen on day 3 after delivery, operational difficulties with the weekend service and staffing problems.

Of the eight women that participated, only one non-smoking participant provided the full complement of samples required in the study (Table 4)—two saliva samples in the antenatal period and collection of all three urine samples from the baby as per the specified time points within the protocol. Overall, four out of eight participants collected a urine sample. This indicates that it was possible for *some* new mothers to collect a sample of baby’s urine by placing cotton wool balls into the nappy and extruding a sample of urine via a syringe. However, the lack of samples collected by three of the participants (only one sample each) indicates that these new mothers needed support and supervision to collect their samples. Support to collect samples of urine in future studies could involve research nurse visits to the house to help support and collect a sample.

It is difficult to draw any generalisable conclusions about the potential to recruit in a future study such as MLASS as the recruitment numbers were so small and the majority of eligible women approached were not interested in participating in the study.

Anecdotal comments from eligible women and their partners at the time they were approached by the researcher and asked to participate in the study suggested that many of the women believed that they already implemented a smoke-free home. Anecdotal comments made by pregnant women and their partners at the recruitment site included “We don’t smoke in the house anyway”, “Not smoking in the house.….it’s common sense isn’t it?” and “Well, if I don’t smoke in my own house then no-one else is going to smoke in it either”.

When we conducted a post hoc survey of pregnant women attending for USS regarding the type of information that pregnant women receive from their community midwife and household smoking restrictions, we found that the majority of pregnant women living in a smoking household who completed the survey believed that they had received SHS-related advice. However, when questioned about the type of advice received, this turned out to be smoking cessation advice. It seems fair to speculate that in this sample of pregnant women, the concept of receiving information about ‘smoke-free homes’ appeared to be associated by the majority of women with any type of information about smoking cessation advice in general. When pregnant women were questioned about household smoking restrictions, the majority of women reported that they already had a smoke-free home before their pregnancy or had implemented smoking restrictions since they had found out they were pregnant.

A small group of women (18 %) indicated in the post hoc survey that there were no smoking restrictions in their house. This indicates that there is a small, potentially hard to reach sub-population of pregnant women who may benefit from a smoke-free homes intervention but who either do not want to engage with any type of smoke-free advice, have not been given any opportunity to discuss such issues with a health professional or have not been able to engage their partner or family members in negotiating smoking restrictions or creating a smoke-free home. This information is important as women who report that there are no smoking restrictions in their house may require targeted identification in any future studies or in routine healthcare practice, rather than attempting to target all pregnant women who smoke or live with a smoker who feel that they will not benefit from the advice or interventions as they are already restricting smoking in their home.

The information regarding the number of women who reported that they already had a smoke-free house either before pregnancy or since pregnancy appears to corroborate anecdotal comments received by the researcher (RM) at the time eligible pregnant women (with their partners) were approached who commented that their homes were already smoke-free and this may also account for the lack of interest by pregnant women in a study that aimed to deliver SFH advice.

However, the findings of the post hoc study have limitations; the post hoc study was based on self-reported information, and therefore, there is no objective validation of whether these respondent’s homes were actually smoke-free. Although respondents may have reported to the researcher either anecdotally during the recruitment phase or completing the post hoc survey that they implemented smoking restrictions, we do not know what being ‘smoke-free’ or having a smoke-free home actually means to these respondents. In addition, the post hoc survey sample was different from those that were approached to participate in the MLASS study, and it is difficult therefore to generalise the findings of the post hoc survey to the wider population of pregnant women who live in smoking households; it is possible that in a different setting, interest in study participation would have been different.

Future investigators could consider changes to the recruitment strategy, changes to the timing and delivery of the intervention and changes to support the women to provide samples to improve the design and methods of the study. For example, the recruitment strategy could be strengthened by the researcher being present in the community clinics at the time the pregnant woman attends her appointments with the community midwife as all pregnant women would have the opportunity to speak to the research team and consider participation—this also avoids approaching non-eligible women, i.e. approaching non-smoking women and also would prevent delays in receiving information about women who may or may not be eligible. In the UK, women’s handheld maternity notes contain standard maternity documentation to record their maternity care. These notes could also contain the first intervention (intervention A) as a matter of usual practice, rather than an extra task to be undertaken during a routine appointment. Community midwives would be able to signpost women to the leaflets during the discussion about smoking status; this would also facilitate brief discussions about secondhand smoke and smoking. At post-delivery discharge from the hospital, women could receive intervention C as part of their routine discharge information/discharge letter rather than on the third postnatal day. The midwife responsible for the new mother’s discharge from the hospital could signpost the new mother to the SHS information as part of her discharge information. Collection of saliva samples from non-smoking pregnant women could be improved if a research assistant or midwifery research support worker could collect the samples at the time the woman attended two routine antenatal appointments at her local GP practice or child health clinic. In the UK, all women usually see their CMW at 16 weeks after the USS confirms pregnancy and have an additional appointment at some point in the third trimester. Dependent on local arrangements and timings of appointments, women would therefore be supported in providing a saliva sample. Collection of urine samples could be improved if a research assistant or midwifery research support worker were based in the CHCs to help women collect the samples. Support to obtain these could be offered at follow-up postnatal visits or where child health care takes place and by changing the postnatal time frame that babies’ urine samples are collected. As part of routine maternity care, women in the UK attend a 6-week postnatal check-up with their GP and are routinely offered the five-in-one infant vaccination (diphtheria, tetanus, whooping cough, Hib and polio) at 8, 12 and 16 weeks old. In addition, health visitors also provide other routine developmental child health clinics, so there is potential opportunity to obtain a urine sample at these appointments.

Supporting participants to provide samples in community clinics would require consideration of capacity of community clinics to host research staff and additional costs of research staff. An additional consideration is that maternity care pathways (i.e. key contact points and care delivery settings) will also vary in different countries; therefore, researchers will need to liaise closely with local maternity services and design future studies that take account of local service configuration to facilitate intervention delivery.

Future studies might also consider inclusion of screening questions, such as those used in the post hoc survey prior to any intervention about current household smoking restrictions in order to target those women who are not currently willing or able to negotiate and implement household smoking restrictions. Consideration of what smoke-free means to pregnant women in order to implement smoking restrictions as part of a targeted intervention should be undertaken. Therefore, qualitative work could be conducted prior to future studies to understand what being smoke-free and a smoke-free home means to pregnant women, what smoke-free homes advice means and the relevance of such advice to pregnant women in early pregnancy.

One of the most challenging aspects of this study was poor recruitment due to lack of interest in the study; therefore, future studies should consider how the study could be designed to be more appealing to pregnant women. It would be useful to conduct qualitative work with another sample of pregnant women and new mothers to obtain feedback on the four interventions used in this study and provide information as to how the interventions can be made more appealing. Furthermore, women in this study did not receive feedback about their cotinine results in pregnancy or the cotinine levels in their baby’s urine. Given that new mothers wish to protect their baby from harms, this may act as motivation to participate in a study and help women identify the level of SHS exposure to their infant.

## Conclusions

In conclusion, due to the small number of participants who participated in the study, it has not been possible to demonstrate the feasibility of collecting relevant data required for an evaluation of a SFH intervention and delivery of a SFH intervention to pregnant women and women with newborn babies. Future recruitment strategies in studies of SFH interventions in the context of routine maternity services and community midwifery service pressures and constraints may need careful consideration and planning in terms of the service capacity to undertake the research, the recruitment setting and method of recruiting pregnant women, the identification of the most appropriate person to deliver the intervention (including timing of delivering the intervention), the criteria for individuals requiring the intervention and individuals’ willingness to engage with SFH research.

## Additional files

Additional file 1:
**Application of a taxonomy of behaviour change techniques used in a smoke-free homes intervention.** (DOCX 23 kb)
Additional file 2:
**Phase 1 focus group discussion (FGD) topic guides and questionnaires.** (DOCX 16 kb)
Additional file 3:
**Interventions A, B, C and D.** (ZIP 1154 kb)
Additional file 4:
**Post hoc survey.** (DOCX 23 kb)
Additional file 5: 
**Adherence by health profesionals to intervention delivery.** (DOCX 17 kb)
