# Supplementary material for: The challenge of mothers learning about secondhand smoke (MLASS): a quasi-experimental, mixed methods feasibility study
Source: Pilot Feasibility Stud. 2016 Feb 6;2:9. doi: 10.1186/s40814-016-0048-0 (PMC5153670; doi:10.1186/s40814-016-0048-0)
Supplement: Additional file 1: — Application of a taxonomy of behaviour change techniques used in a smoke-free homes intervention. (DOCX 23 kb) [file 40814_2016_48_MOESM1_ESM.docx]

Additional file 1: Application of a Taxonomy of Behaviour Change Techniques used in a Smoke Free Homes Intervention

Analysis of the activities delivered in the smoke free homes intervention and their links to the 26 Behaviour Change Techniques which have been identified in the paper ‘A Taxonomy of Behaviour Change Techniques Used in Interventions’ (2008) Abrahams and Michie - Health Psychology 2008 Vol.27, No.3, 379-387

The activities listed could be used in 2 scenarios

1. Health Worker → Pregnant woman (non smoker)
2. Pregnant woman→ Smoking partner / family

| BCT | Determinant/  Domain (Michie) | Link to theoretical frame work | Definition | Activity suggestions / comments | | Agent | | Recipient |
| --- | --- | --- | --- | --- | --- | --- | --- | --- |
| 1. Provide information about behaviour health link | Attitude  Knowledge  Beliefs about consequences  Motivation and goals *(confidence building)* | Information-motivation-behavioural skills model (IMB) | General information about behavioural risk, for example susceptibility to poor health outcomes or mortality risk in relation to the behaviour | Positive messages about benefits | | HW | | Pregnant Woman |
|  |  |  |  |  | | Pregnant Woman | | Partner / Family |
| 2. *Explore* information on consequences  *(discussion around consequence)* | Motivation | Theory of Reasoned Action (TRA)  Theory of Planned Behaviour (TPB) Social –cognitive theory (SCogT) | Information about the benefit and costs of action or inaction, focusing on what will happen if the person does or does not perform the behaviour | Identification of perceived cost benefits balance in relation to the discussion with partner | | HW | | Pregnant Woman |
|  | motivation |  |  | Identification of perceived cost benefits balance in relation to the baby | | Pregnant Woman | | Partner / Family |
|  |  |  |  |  | |  | |  |
| 3. Provide information about others’ approval | Social influences  Motivation  Belief about capabilities | (TRA, TPB, IMB) | Information about what others think about the person’s behaviour and whether others will approve or disapprove of any proposed behaviour change | Present information on social norms (most babies live in SFHs ) but understand and take into account the immediate social norm of the mother and the family – using examples of other women who have discussed at home and achieved SFH | | HW | | Pregnant Woman |
|  | Social influences  Motivation  Belief about capabilities |  |  | Present information on social norms (most babies live in SFHs as many people have stopped smoking) but understand and take into account the immediate social norm of the family. Use as a motivational tool for goal setting | | Pregnant Woman | | Partner / Family |
| 4. Prompt intention formation | motivation | (TRA, TPB, SCogT, IMB) | Encouraging the person to decide to act or set a specific goal, for example, to make a behavioural resolution such as “I will take more exercise next week.” | Intention – have the discussion with the partner  Mother to choose a goal they are confident they can achieve re. raising the issue with partner / family  ‘DO you believe that having the conversation will lead to a change?’  ‘Are you confident that you could have the discussion with your partner?’ | HW | | Pregnant Woman | |
|  |  |  |  | Is the intention to have a SFH  ‘How important is it for the family / baby to have a SFH’?  ‘How confident are they this can be achieved?’ | Pregnant Woman | | Partner / Family | |
| 5. Prompt barrier identification |  | (SCogT) | Identify barriers to performing the behaviour and plan ways of overcoming them | Explore barriers to having the discussion and a SFH (e.g. does the family live in a flat? Does the mother live in their own home or staying with someone else where they feel they may not be in a position to raise the issue?) | | HW | | Pregnant Woman |
|  |  |  |  | Explore barriers to having a SFH | | Pregnant Woman | | Partner / Family |
| 6. Provide general encouragement | Motivation  attitude | (SCogT) | Praising or rewarding the person for effort or performance without this being contingent on specified behaviours or standards of performance | Praise the intention and provide positive encouragement - building rapport | HW | | Pregnant Woman | |
|  |  |  |  | Praise the intention and provide positive encouragement - building family engagement and praising resolve to go SF | Pregnant Woman | | Partner / Family | |
| 7. Set graded tasks | Skills  Motivation  knowledge | (SCogT) | Set easy tasks and increase difficulty until target behaviour is performed | Encourage woman to identify small steps towards the goal of having the discussion about SFHs. May want to discuss some suggestions and encourage the women to come up with her own … e.g. leaving a leaflet on a table, are there any TV ads that could prompt a discussion? | HW | | Pregnant Woman | |
|  |  |  |  | Could use SFH steps and 4 week challenge – smoke away from baby – smoke in one room only – totally SFH – stay smoke free for at least 4 weeks | Pregnant Woman | | Partner / Family | |
| 8. Provide instruction |  | (SCogT) | Telling the person how to perform a behaviour and/or preparatory behaviours | NA | | HW | | Pregnant Woman |
| 9. Model or demonstrate the behaviour / *Rehearsal of relevant skills* | Skills  Social influence | (SCogT) | An expert *explores with the person/* shows the person how to correctly perform a behaviour, for example, in class or on video | ? demo of discussion / role play / Able to practise | | HW | | Pregnant Woman |
|  |  |  |  |  | | Pregnant Woman | | Partner / Family |
| 10. Prompt specific goal setting | Self efficacy  Skills  motivation | Control theory (CT) | Involves detailed planning of what the person will do, including a definition of the behaviour specifying timeframe, scope or duration and specification of at least one context, that is where, when, how, or with whom | Encourage woman to develop strategy e.g. Identify appropriate time to have discussion. Possibly develop SMART objective | HW | | Pregnant Woman | |
|  |  |  |  | Agree specific goal and steps along the way. Set SMART objectives for various steps. E.g. set a 4 week smoke free challenge | Pregnant Woman | | Partner / Family | |
| 11. Prompt review of behavioural goals |  | (CT) | Review and/or reconsideration of previously set goals or intentions | Discussion – how did it go?  And ‘what else?’ | HW | | Pregnant Woman | |
|  |  |  |  | How do we feel about this – is it working for us? | Pregnant Woman | | Partner / Family | |
| 12. Prompt self-monitoring of behaviour | Skills  Attitude | (CT) | The person is asked to keep a record of specified behaviour(s) (e.g. in a diary) | N/A | HW | | Pregnant Woman | |
|  |  |  |  | Possibly use a monitoring tool e.g. diary / sticker chart | Pregnant Woman | | Partner / Family | |
| 13. Provide feedback on performance | Attitude  Beliefs about consequences and capabilities | (CT) | Providing data about recorded behaviour or evaluating performance in relation to a set standard or others’ performance i.e. the person received feedback on their behaviour | Asking what worked well / was didn’t work so well / what could have been different | HW | | Pregnant Woman | |
|  |  |  |  | How did the family perform in relation to steps / SMART objectives – could use diary / sticker chart … what has worked well – if we didn’t achieve what could we change | Pregnant Woman | | Partner / Family | |
| 14. Provide contingent rewards | Skills  motivation | Operant conditioning (OC) | Praise, encouragement that are explicitly linked to the achievement of specified behaviours | Praise if successful discussion and encourage to use alternative strategies if not gone so well | HW | | Pregnant Woman | |
|  |  |  |  | Goody bag?  Identify the positives e.g. house smells better, baby is going to be healthier, maybe smoking less therefore saving money | Pregnant Woman | | Partner / Family | |
| 15. Teach to use prompts or cues | Memory, attention, decision process and action planning | (OC) | Teach the person to identify environmental cues that can be used to remind them to perform a behaviour, including times of day or elements of contexts | e.g. no smoking signs, removing ashtrays, umbrella by door - agree which are acceptable | HW | | Pregnant Woman | |
|  |  |  |  |  | Pregnant Woman | | Partner / Family | |
| 16. Agree on behavioural contracts | Motivation  Action planning | (OC) | Agreement (i.e. signing) of a contract specifying behaviour to be performed so that there is a written record of the person’s resolution witnessed by another | N/A | HW | | Pregnant Woman | |
|  |  |  |  | Could be family agreement e.g. signing a SFH pledge … Smoking fine if not done outside | Pregnant Woman | | Partner / Family | |
| 17. Prompt practice |  | (OC) | Prompt the person to rehearse and repeat the behaviour or preparatory behaviours | Encourage women to rehearse discussion beforehand | HW | | Pregnant Woman | |
|  |  |  |  | Maybe think about dealing with specific situations / people e.g. friends visiting | Pregnant Woman | | Partner / Family | |
| 18. Use follow up prompts | Self efficacy |  | Contacting the person again after the main part of the intervention is complete | Asking about SFH at routine visits | HW | | Pregnant Woman | |
|  |  |  |  | N/A | Pregnant Woman | | Partner / Family | |
| 19. Provide opportunities for social comparison | Social influences | (SCompT) | Facilitate observation */ raising awareness*  of non-expert others’ performance for example, in a group class or using video or case study | Depends on context … could be a group intervention in e.g. children’s centre | HW | | Pregnant Woman | |
|  |  |  |  | Maybe visit friends / family who are smoke free or thinking about how many people the family know who are smoke free | Pregnant Woman | | Partner / Family | |
| 20. Plan social support or social change | Social influences | (Social support theories) | Prompt consideration of how others could change their behaviour to offer the person help or (instrumental) social support, including “buddy” systems and/or providing social support | Antenatal group activity / buddying up | HW | | Pregnant Woman | |
|  |  |  |  | Partner / friend supporting the mother discuss SFH – possibly backing up or helping enforce / having the discussion on behalf of the mother | Pregnant Woman | | Partner / Family | |
| 21. Prompt identification as a role model |  |  | Indicating how the person may be an example to others and influence their behaviour or provide an opportunity for the person to set a good example | Positive example of tackling a sensitive issue | HW | | Pregnant Woman | |
|  |  |  |  | Wanting to be perceived as being good parents and role model to prevent children taking up smoking in later life | Pregnant Woman | | Partner / Family | |
| 22. Prompt self-talk | Beliefs about capabilities |  | Encourage the use of self-instruction and self-encouragement (aloud or silently) to support action | ? | | HW | | Pregnant Woman |
| 23. Relapse prevention | Self efficacy  Skills  Beliefs about capabilities | (Relapse prevention therapy) | Following initial change, help identify situations likely to result in readopting risk behaviours or failure to maintain new behaviours and help the person plan to avoid or manage these situations | N/A | HW | | Pregnant Woman | |
|  |  |  |  | Identifying the possible triggers that could result in smoking in the house – and strategies to address them | Pregnant Woman | | Partner / Family | |
| 24. Stress management | Self efficacy | (Stress theories) | May involve a variety of specific techniques (e.g. progressive relaxation) that do not target the behaviour but seek to reduce anxiety and stress | ? | HW | | Pregnant Woman | |
|  |  |  |  | ? | Pregnant Woman | | Partner / Family | |
| 25. Motivational interviewing | Motivation  Beliefs about capabilities |  | Prompting the person to provide self-motivating statements and evaluations of their own behaviour to minimise resistance to change | ? | HW | | Pregnant Woman | |
|  |  |  |  | ? | Pregnant Woman | | Partner / Family | |
| 26. Time management |  |  | Helping the person make time for the behaviour (e.g. to fit it into a daily schedule) | N/A | HW | | Pregnant Woman | |
|  |  |  |  | N/A | Pregnant Woman | | Partner / Family | |
