# Supplementary material for: The challenge of mothers learning about secondhand smoke (MLASS): a quasi-experimental, mixed methods feasibility study
Source: Pilot Feasibility Stud. 2016 Feb 6;2:9. doi: 10.1186/s40814-016-0048-0 (PMC5153670; doi:10.1186/s40814-016-0048-0)
Supplement: Additional file 2: — Phase 1 focus group discussion (FGD) topic guides and questionnaires. (DOCX 16 kb) [file 40814_2016_48_MOESM2_ESM.docx]

**Additional file 2: Phase 1 Focus Group Discussion (FGD) Topic Guides and Questionnaires**

**FGD topics with new mother’s**

Have you heard of SHS?

Were you given information during pregnancy?

When should you be given information about SHS?

What would you want to read about or hear to help you reduce second-hand smoke?

Let’s look at some materials about SHS produced previously… What ideas do you have for materials/products with slogans?

**FGD topics with health professionals**

What are the key messages about SHS that parents need to be told?

What are the key message about SHS that others (such as friends, relatives) need to be told?

**Phase 1 development work: women’s questionnaire**

**Are you a new mum?**

**Are you an expectant mum?**

**Do you know when a baby is affected by second-hand smoke?**

***(Respondents were asked to tick all statements they knew)***

a. When someone smokes in the same room as the baby

b. When someone smokes in one room when the baby is asleep in another

c. When someone picks up your baby but hasn’t washed their hands after having a cigarette

d. When the baby is taken in a car someone has just been smoking in

e. When a baby touches clothes, sofa or carpets in a room where people have smoked

**Second-hand smoke either causes or can increase the risk of these**

**(*Respondents were asked to tick as many as they knew)***

a. Asthma

b. Lung infections

c. Glue ear

d. Cradle cap

e. Sudden infant death

f. Meningococcal disease *(blood disease)*

g. Increased hospital visits

h. Low birth weight

i. Babies which are small for age in womb

j. Skin rashes

k. Wheezing

**What would be the best way to help you reduce second-hand smoke for your baby?** *(Respondents were asked to prioritise which would be most useful); 1 being most useful and 5 being least*

a. Support on how to influence smokers in your family, so that they don’t smoke around your baby

b. The Facts about the chemical content of cigarette smoke such as arsenic, lead and cyanide

c. The Facts that when someone smokes, the toxic chemicals from cigarettes coat clothing and stay on your carpets and soft

d. To understand adults can choose but baby has no choice

e. To know that even if me or my family smoke, we know what we can do to keep the baby safe

**When would it be good to get this information? *(Respondents were asked to tick the most useful time)***

a. On booking in with the midwife

b. At six months pregnant

c. 2 weeks before the birth due date

d. 2 weeks after the birth

e. 3 months after

**Phase 1 development work: health professional’s questionnaire**

**What information do you think the intervention material should include?** *Respondents were asked to priorities their selections, with 1 as most important*

a. Facts about the chemicals SHS contains eg arsenic, ammonia

b. Facts about the impact on babies’ health, eg respiratory problems, glue ear

c. How smoke coats furnishings and clothes for hours after the cigarette is out

d. How SHS in cars is particularly harmful for babies

e. That the baby has no choice

f. The positive steps they can take to make the baby’s space as smoke free as possible

g. How they may influence relatives’ and friends’ smoking behaviour, as well as their own

**When do you think it would be best to give this information?** *This could be once, or more than once*

a. At a routine ante-natal appointment – please say which visit and by whom

b. At a routine post-natal appointment – please say which visit and by whom

**Which health professional do you think might be best placed to give the intervention material?**

a. GP

b. Midwife

c. Midwifery Support Worker

d. Health Visitor

e. Health Visitor Support Worker

f. Other (*please say)*

**What does the Health Professional need to say when they give the intervention?** *Respondents were asked to tick all they thought would be useful.*

a. I need to tell you about the harm SHS may do to your baby

b. Here is something local women have found useful

c. Do you have any relatives or friends who smoke who will come to see the baby? We appreciate it’s hard to ask them not to smoke, so here’s something which may help

d. Any reduction in your baby’s exposure to SHS is better than none

e. All Health Professionals give praise for implementing any change, however small

**What media/method is best?** *(Respondents were asked to prioritise which would be most valuable, with 1 as most valuable)*

a. A leaflet in the back of the Red Book

b. A catchy slogan e.g. you can choose, baby can’t; we don’t smoke near baby

c. A bag, with slogan, to put their Maternity Notes in

d. Posters in ante-natal settings

e. A sticker for house and car

A fridge magnet

g. Post-its and pencils

h. Tea towels

i. Birth announcement cards, with message, to give to relatives and friends which Mum can personalise, personalised

j. Multiple options of the above

k. Other – please give details

**Have you ever spoken to a woman about SHS?**

**FGD Topic guide: design of intervention women and health professionals**

Number of items Item/ phrases

What type of device?

What does it say?

What does the Midwife say when she gives it?
