# Supplementary material for: The challenge of mothers learning about secondhand smoke (MLASS): a quasi-experimental, mixed methods feasibility study
Source: Pilot Feasibility Stud. 2016 Feb 6;2:9. doi: 10.1186/s40814-016-0048-0 (PMC5153670; doi:10.1186/s40814-016-0048-0)
Supplement: Additional file 3: — Interventions A, B, C and D. (ZIP 1154 kb) [file 40814_2016_48_MOESM3_ESM.zip › Appendix 3_Intervention 1R2.pdf]

NHS

This bubble will appear blank, or white, until touched to activate thermchromatic ink

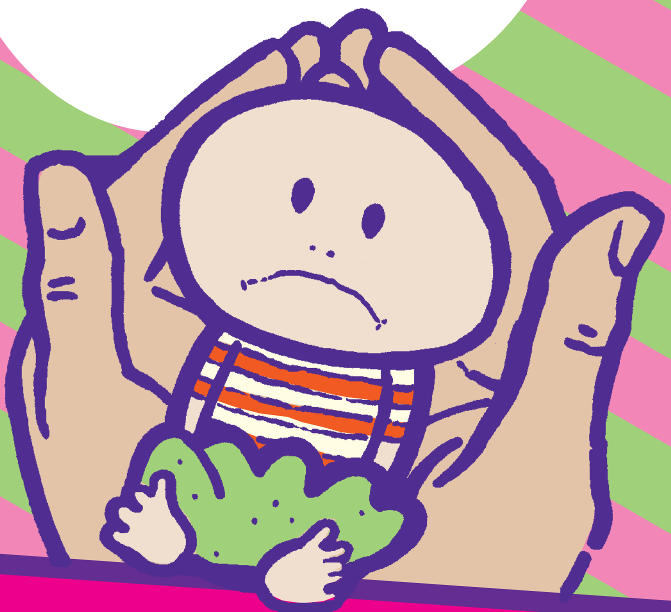

Second Hand Smoke

is the smoke which comes from other people's cigarettes. The smoke is full of chemicals and poisons, including lead, arsenic and cyanide.

Though you can't see it or smell it, it is like invisible grey snow, falling silently onto furniture and carpets in rooms where people smoke. Because your baby relies on the air you breathe to develop and grow, if you breathe in smoke, so does your growing baby.

PROTECT me... From Second Hand Smoke

DEVICE 1 A4.indd 1 16/3/12 14:33:53

NHS

SECOND HAND SMOKE IS AN INVISIBLE THREAT

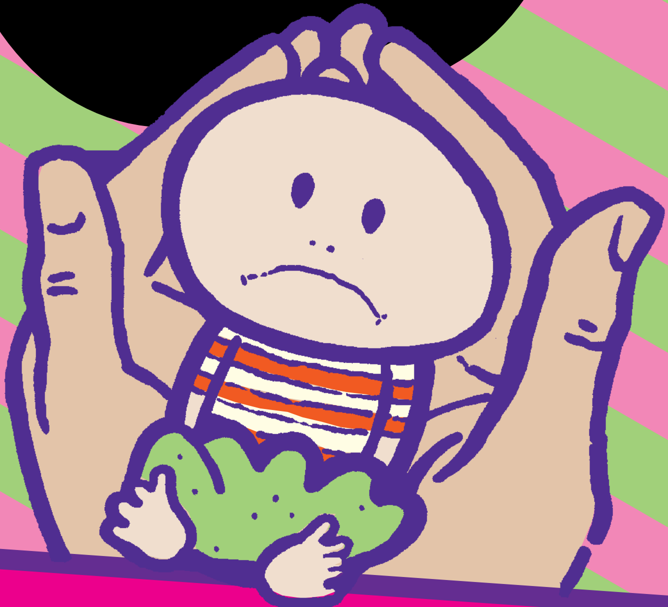

Second Hand Smoke

is the smoke which comes from other people's cigarettes. The smoke is full of chemicals and poisons, including lead, arsenic and cyanide.

Though you can't see it or smell it, it is like invisible grey snow, falling silently onto furniture and carpets in rooms where people smoke. Because your baby relies on the air you breathe to develop and grow, if you breathe in smoke, so does your growing baby.

PROTECT me...

From Second Hand Smoke

DEVICE 1 A4.indd 2 16/3/12 14:33:53

SECOND HAND SMOKE IS AN INVISBLE THREAT

Can you answer these questions?
Are they true or false? (reveal the answers)

Q: What is Second Hand Smoke?

A: Smoke from the burning end of a cigarette — TRUE

A: Smoke blown out by someone smoking — TRUE

A: Smoke still in a room when people have stopped smoking — TRUE

Q: How does Second Hand Smoke affect baby?

A: Chemicals you breathe in with SHS go into baby's blood — TRUE

A: Baby gets less oxygen to grow — TRUE

A: Baby is more at risk of many health problems — TRUE

Do *whatever* you feel you can to keep your growing baby safe!

- Ask smokers not to light up in your home
- If someone has smoked, open the windows and doors to get rid of smoke before you sit in the room
- Move away from people smoking in the street

Remember, if you breathe in smoke, so does your growing baby.

Protect me... From Second Hand Smoke

NHS

DEVICE 1 A4.indd 3 16/3/12 14:33:54
