# Supplementary material for: The challenge of mothers learning about secondhand smoke (MLASS): a quasi-experimental, mixed methods feasibility study
Source: Pilot Feasibility Stud. 2016 Feb 6;2:9. doi: 10.1186/s40814-016-0048-0 (PMC5153670; doi:10.1186/s40814-016-0048-0)
Supplement: Additional file 3: — Interventions A, B, C and D. (ZIP 1154 kb) [file 40814_2016_48_MOESM3_ESM.zip › Appendix 3_Intervention 2R2.pdf]

# SECOND HAND SMOKE IS AN INVISIBLE THREAT

PROTECT me...

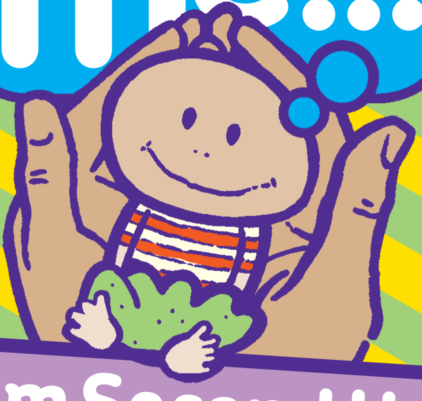

From Second Hand Smoke

NHS

DEVICE 2 A5.indd 1 16/3/12 14:36:56

# SECOND HAND SMOKE IS AN INVISIBLE THREAT

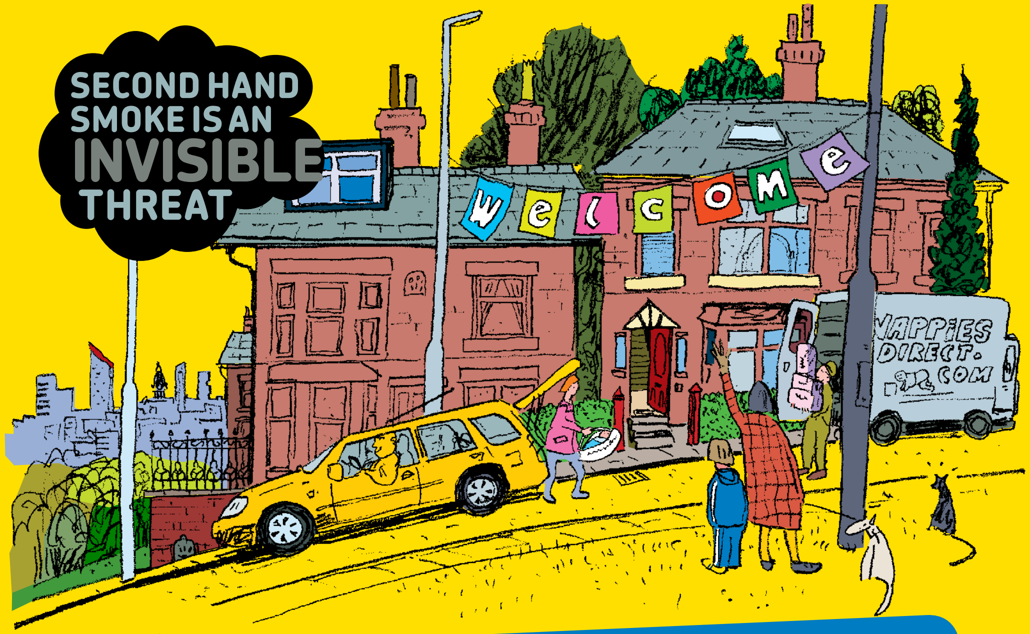


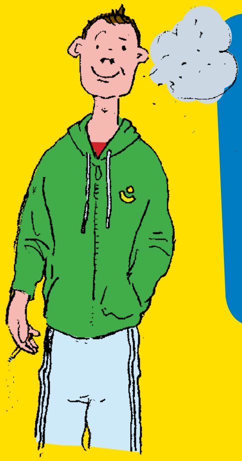

Soon you'll be welcoming your new baby into the world. Because your baby depends on you to stay safe and well, we need to let you know about an invisible danger which may be in your home and which could seriously harm Baby while still tiny; and also what you can do to protect your baby.

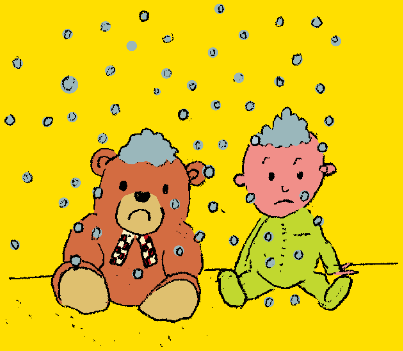

Second Hand Smoke is the smoke which comes from the burning end of a cigarette and the smoke blown out by smokers. The smoke is full of chemicals and poisons, including arsenic, lead and cyanide. Though you can't always see these chemicals, they are like invisible grey snow, falling silently onto furniture and carpets in rooms where people smoke. They stick on your hands and clothing and rub off onto the things you touch–including baby–even some time after stubbing out the cigarette. Your baby depends on you to help keep those tiny lungs safe.

**If you, a friend or relative, smokes in the room with Baby, then Baby breathes in carbon monoxide, cyanide, arsenic and lead.**

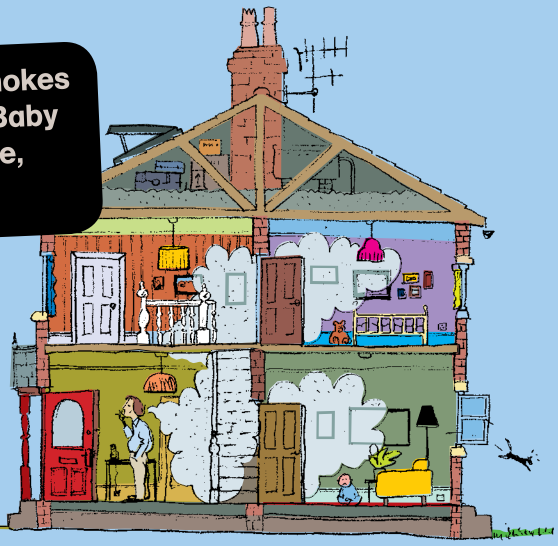

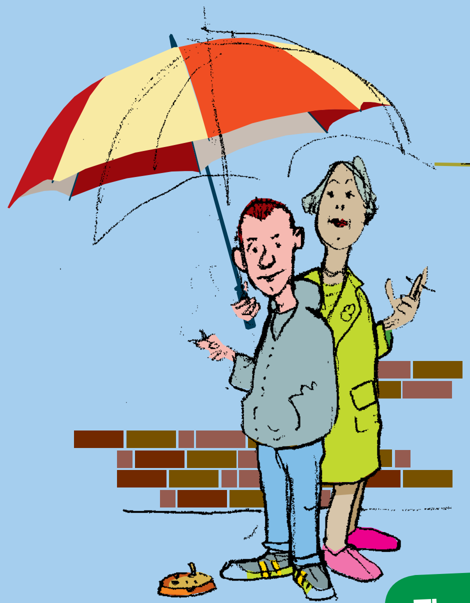

If someone who smokes picks up your baby, the smoke chemicals from their clothes will rub onto the baby, who will then transfer them to their mouth!

Baby is more likely to get asthma, chest infections and other illnesses

Babies in homes where people regularly smoke in the house are more likely to die suddenly

**There is a lot you can do to reduce the risk of harm as much as possible, or even remove the risk completely.**

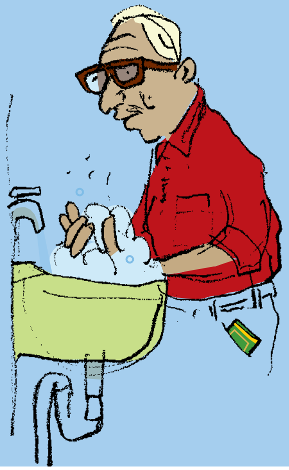

**If you smoke, pop outside**—keep an ashtray, an umbrella and a 'smoking jacket' by the door! **Then always wash your hands after your cigarette.**

**Ask your friends and relatives to have their last cigarette as long as possible before visiting the baby.**

**If they want to smoke at your house, ask them to pop outside to smoke and then wash their hands when they come back in.**

DEVICE 2 A5.indd 2-3 16/3/12 14:36:57

# THINKING OF GIVING UP?!?

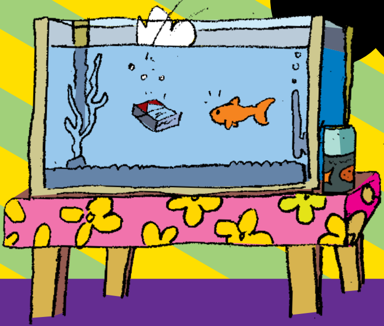

You—or your friends or relatives—might want to take the opportunity to stop smoking when Baby arrives; if you do, the NHS can provide support for you all—why not stop together?

**Call 0800 169 4219 for Leeds NHS Stop Smoking Service.**

PROTECT me... From Second Hand Smoke

NHS
