# Supplementary material for: The challenge of mothers learning about secondhand smoke (MLASS): a quasi-experimental, mixed methods feasibility study
Source: Pilot Feasibility Stud. 2016 Feb 6;2:9. doi: 10.1186/s40814-016-0048-0 (PMC5153670; doi:10.1186/s40814-016-0048-0)
Supplement: Additional file 3: — Interventions A, B, C and D. (ZIP 1154 kb) [file 40814_2016_48_MOESM3_ESM.zip › Appendix 3_Intervention 3R2.pdf]

COVER MAGNET

HELLO!

WRITE NAME [illegible]RE:

Granny

PROTECT me...

From Second Hand Smoke

I'm finally here! I'd love to see you, but there is something I need to let you know...

NHS

DEVICE 3.indd 1 16/3/12 15:09:18

Pg1 of printed 10pp leaflet

Blank: magnet cover back sticks here

Second Hand Smoke is the smoke which comes from the burning end of a cigarette and the smoke blown out by smokers. The smoke is full of ***chemicals*** and ***poisons***, including arsenic, lead and cyanide.

SECOND HAND SMOKE IS AN INVISIBLE THREAT

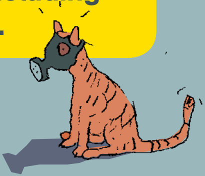

DEVICE 3.indd 3 16/3/12 15:09:18

Though you can't *see* the chemicals, they are like invisible grey snow, falling silently onto furniture and carpets in rooms where people smoke. They stick on your hands and clothing and rub off onto the things you touch—including me—even some time after stubbing out the cigarette.

I DEPEND ON YOU TO HELP KEEP MY TINY LUNGS SAFE!

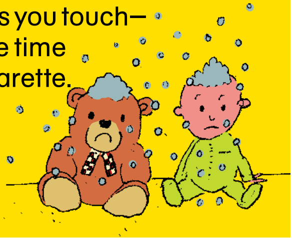

If you smoke in the room with me, then I breathe in carbon monoxide, cyanide, arsenic, lead and other *bad things*. The smoke chemicals will *coat* the carpets and sofas.

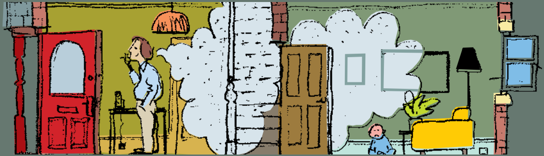

DEVICE 3.indd 5 16/3/12 15:09:18

If anyone picks me up after having a cigarette, the ***smoke chemicals*** from their clothes will rub onto me—and then I'll put my fingers in my mouth!

I'm more likely to get asthma, chest infections and other illnesses.

I could even DIE—and I've only just got here!

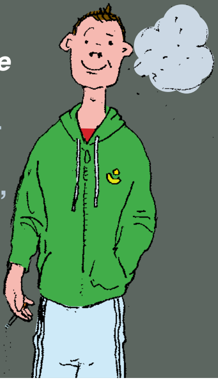

BACK COVER MAGNET

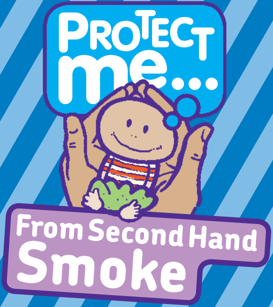

NHS

DEVICE 3.indd 7 16/3/12 15:09:18

Protect me...

From Second Hand Smoke

WHAT YOU CAN DO...

DEVICE 3.indd 8 16/3/12 15:09:19

Have your *last* cigarette
as long as possible
before visiting me...

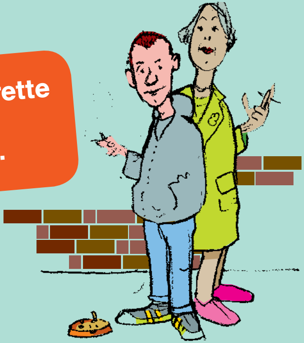

DEVICE 3.indd 9 16/3/12 15:09:19

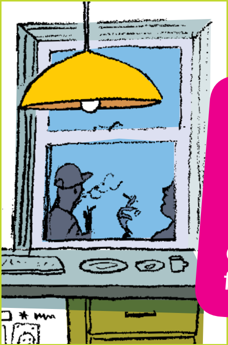

Please don't smoke in the house at all, my lungs are so small they soon fill up with smoke– pop outside, my mum can help with an ashtray for you.

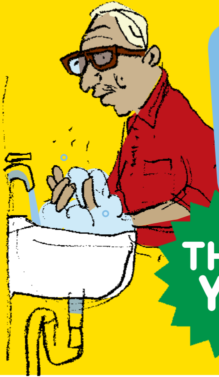

Give your hands a quick **wash** before picking me up. Ask my other relatives and mum's friends not to smoke around me either.

THANK YOU

FOR PROTECTING ME FROM SECOND HAND SMOKE!

Pg 10 of printed
10pp leaflet
Blank: magnet cover
back sticks here
