# Supplementary material for: The challenge of mothers learning about secondhand smoke (MLASS): a quasi-experimental, mixed methods feasibility study
Source: Pilot Feasibility Stud. 2016 Feb 6;2:9. doi: 10.1186/s40814-016-0048-0 (PMC5153670; doi:10.1186/s40814-016-0048-0)
Supplement: Additional file 3: — Interventions A, B, C and D. (ZIP 1154 kb) [file 40814_2016_48_MOESM3_ESM.zip › Appendix 3_Intervention 4R2.pdf]

Protect me...

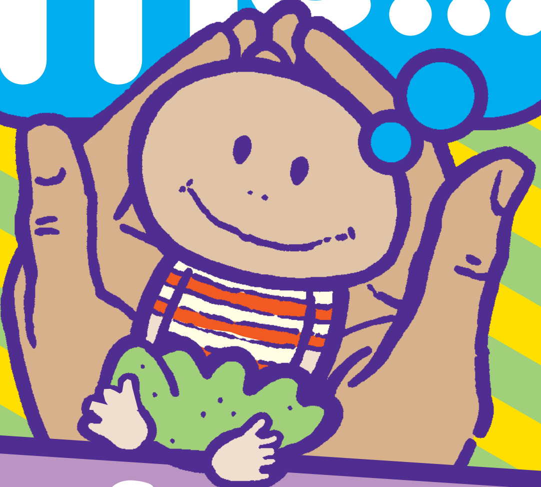

From Second Hand Smoke

NHS

DEVICE 4 A5 .indd 1 2/4/12 10:39:27

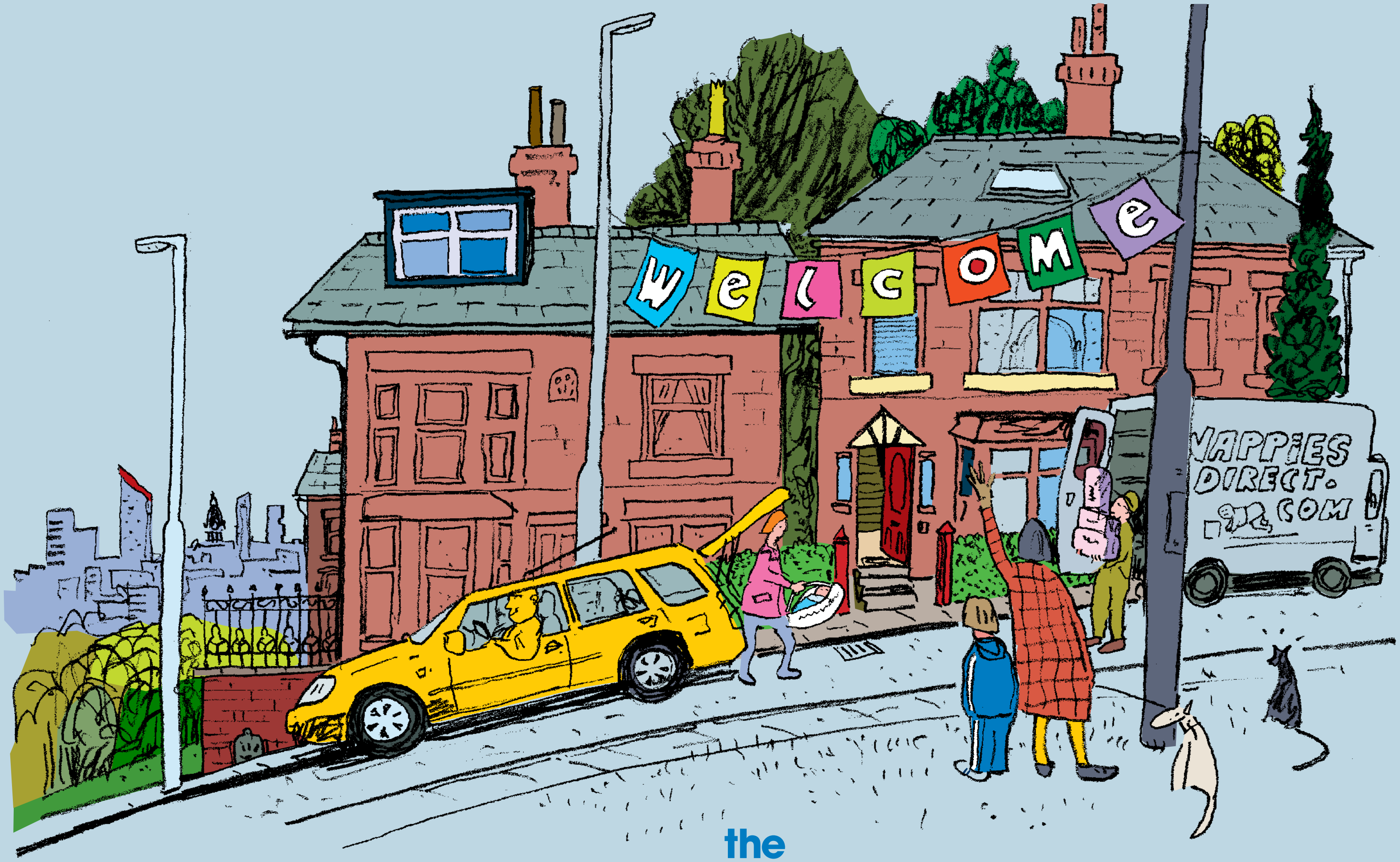

the HomeComing

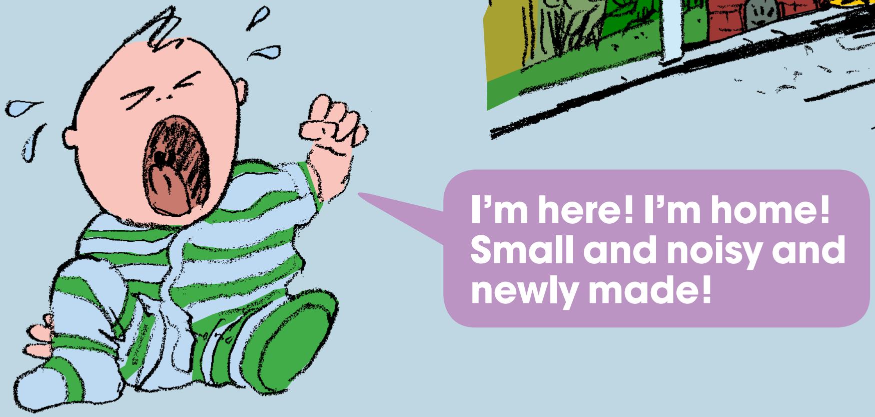

DEVICE 4 A5 .indd 2-3 2/4/12 10:39:28

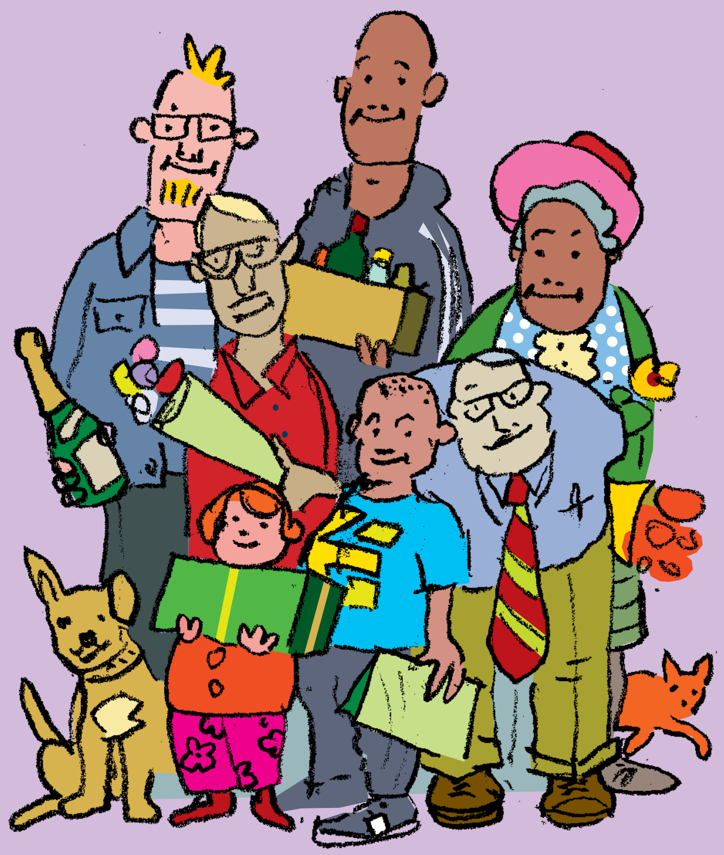

I'd like to welcome *all* my visitors.

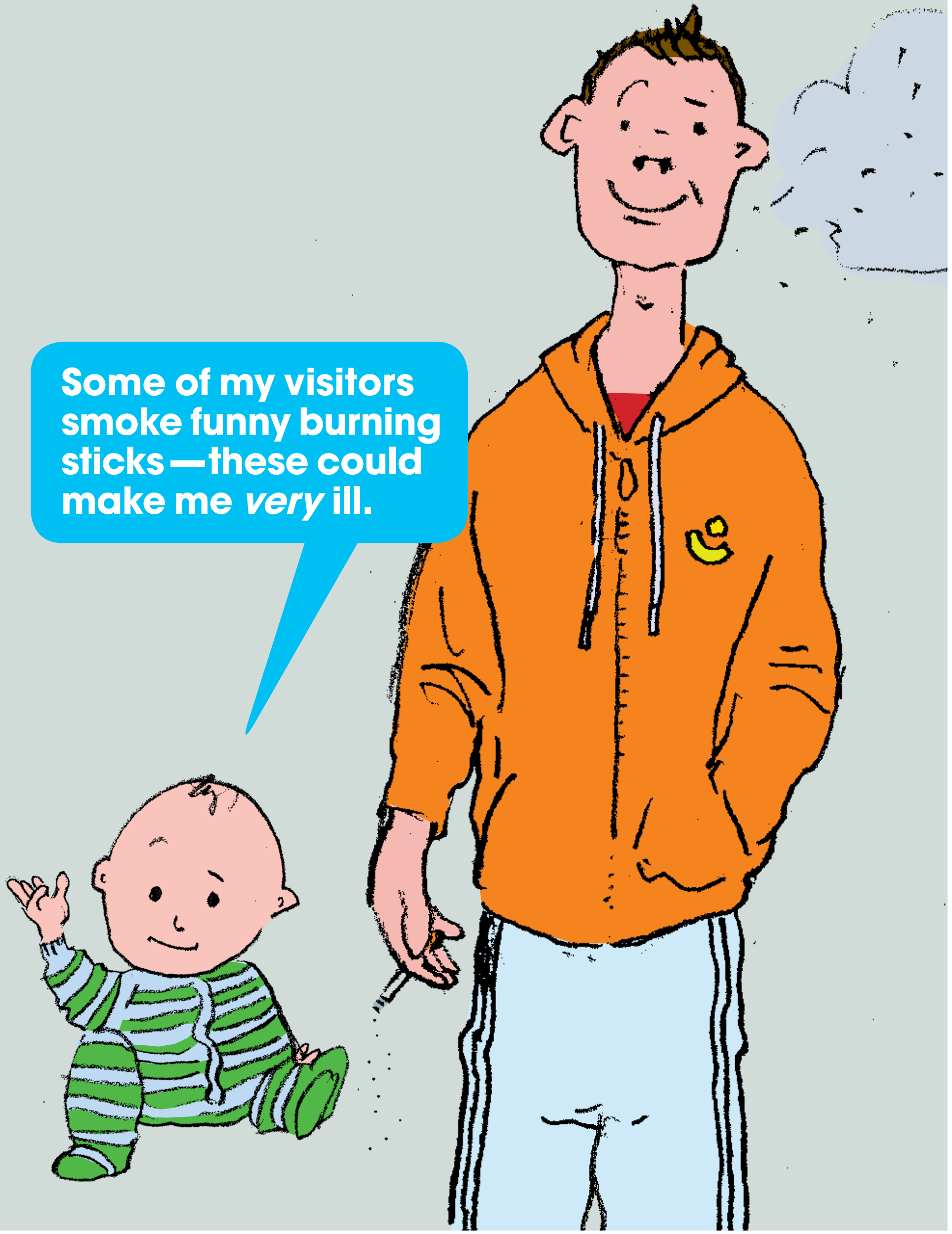

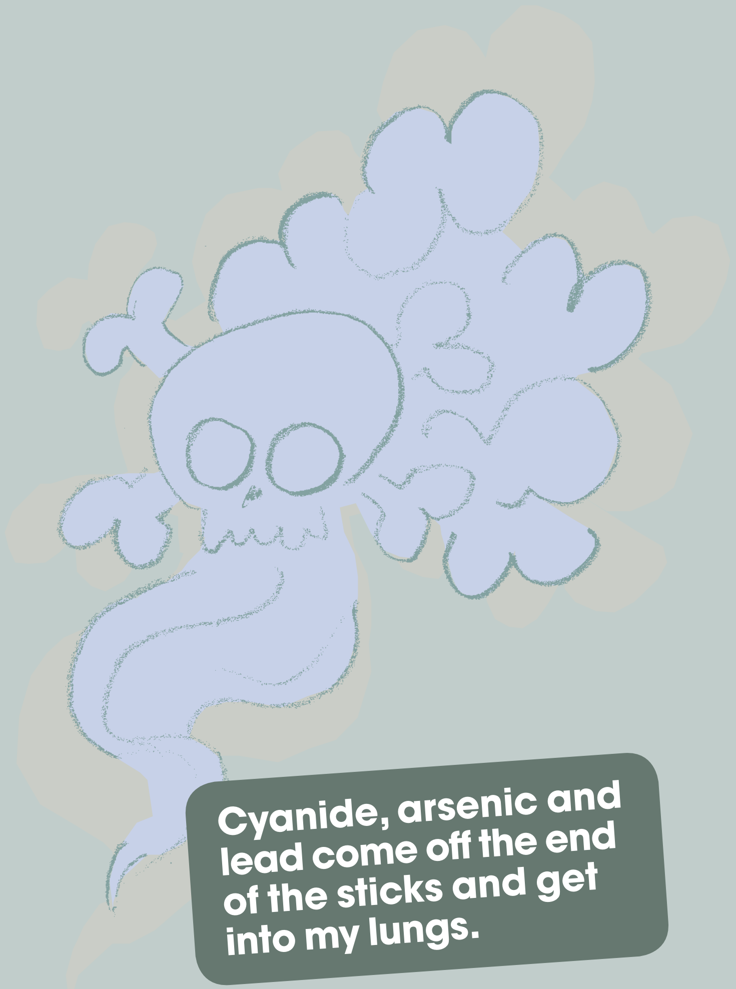

Cyanide, arsenic and lead come off the end of the sticks and get into my lungs.

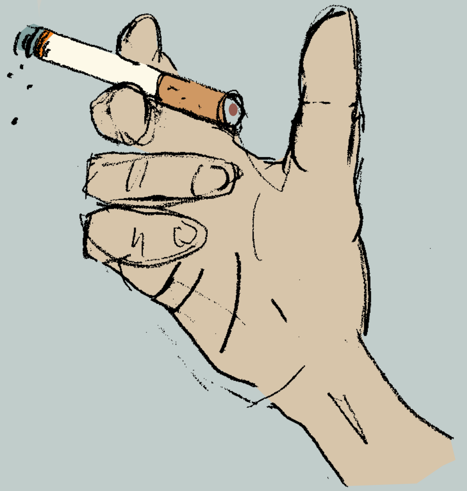

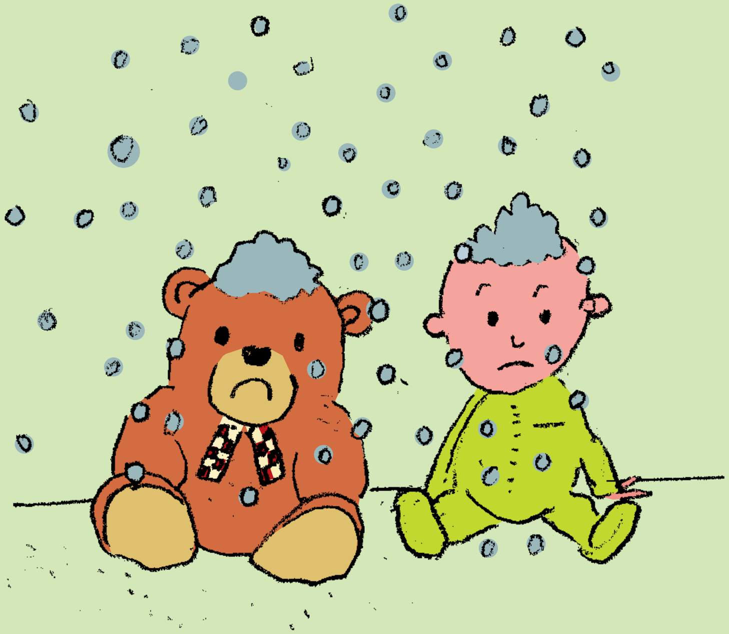

Bits float in the air and coat the carpets and sofas, like invisible grey snow.

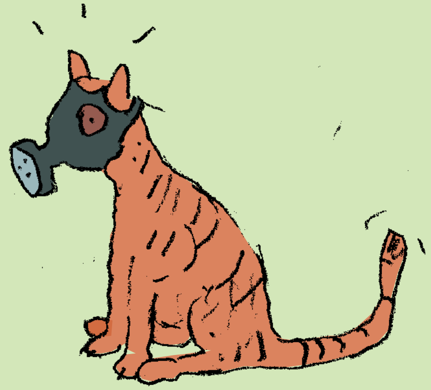

DEVICE 4 A5 .indd 6-7 2/4/12 10:39:29

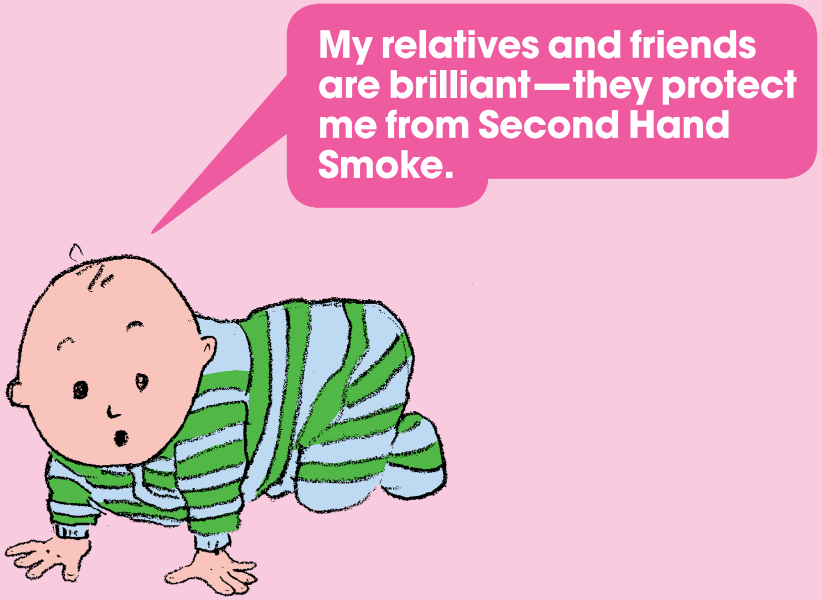

So Aunty Meg has thrown her cigs away...

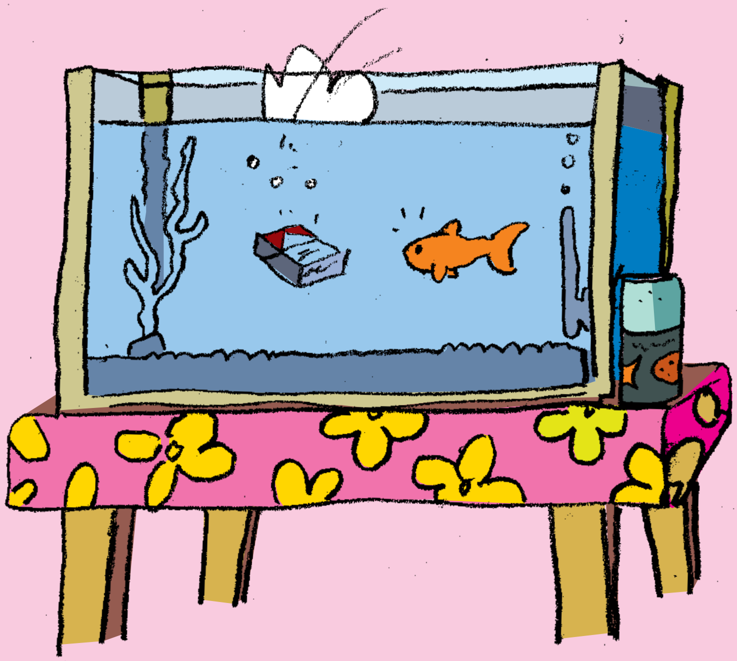

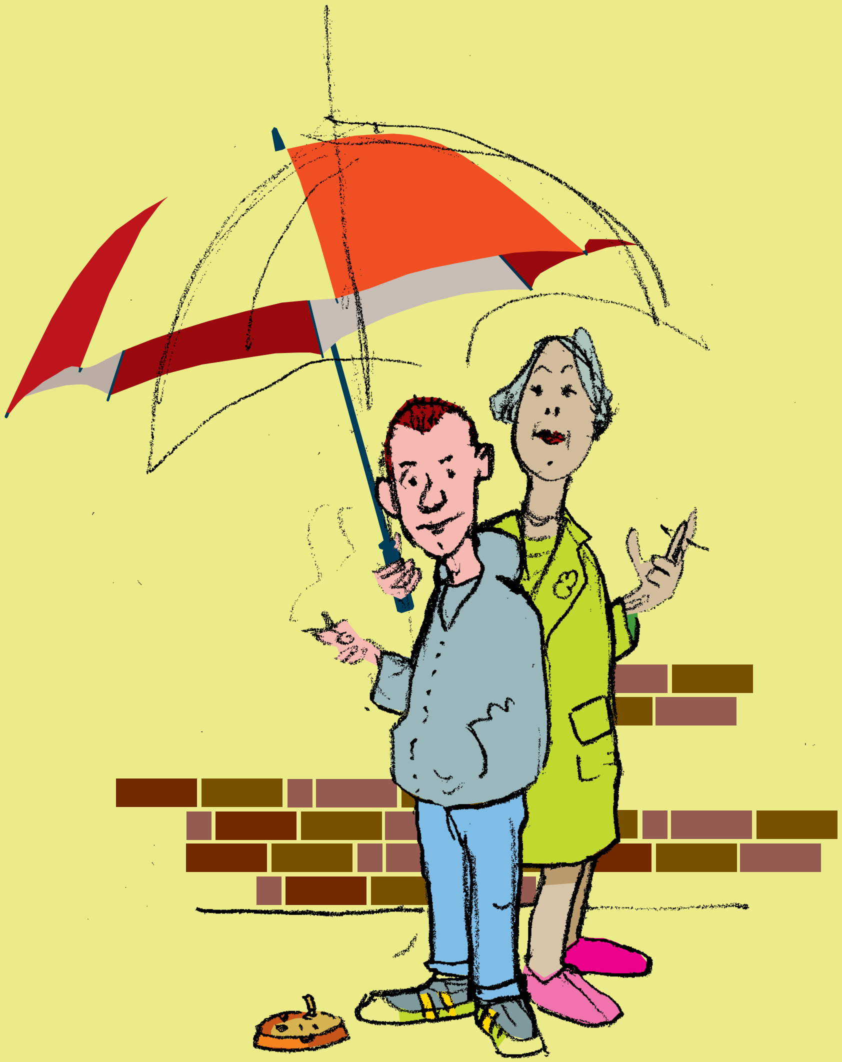

...Kev and Reina go outside so my little lungs don't fill up with smoke.

There's an ashtray and an umbrella at the back door in case it rains. Grandma laughs, saying "Stop smoking and save your money for a holiday!"

DEVICE 4 A5 .indd 8-9 2/4/12 10:39:29

Mum has locked all cigarettes away. Our visitors are brilliant. They only smoke outside.

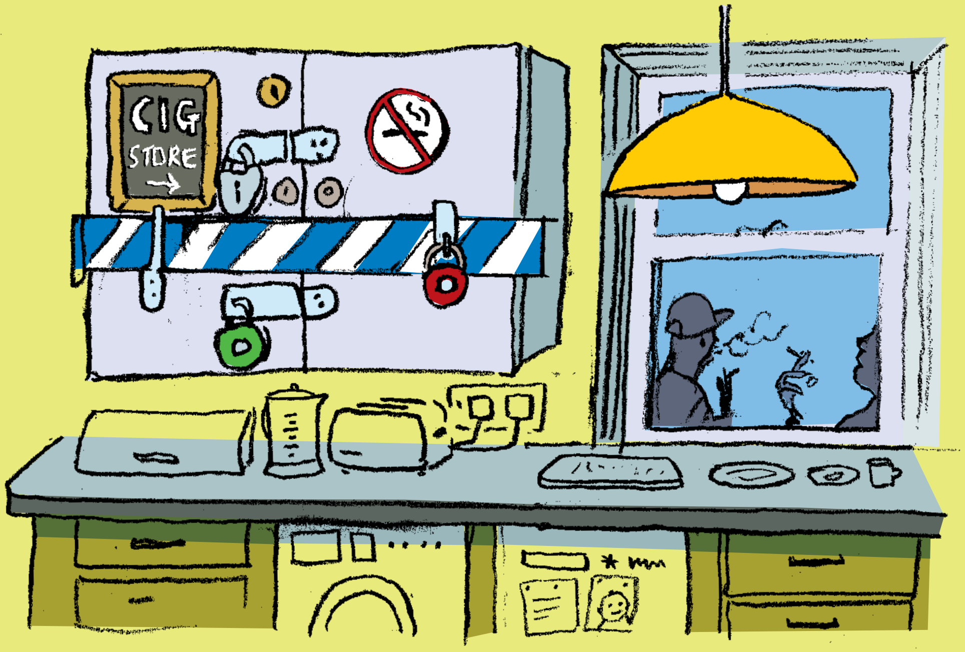

Our neighbour Hardeep sometimes smokes.

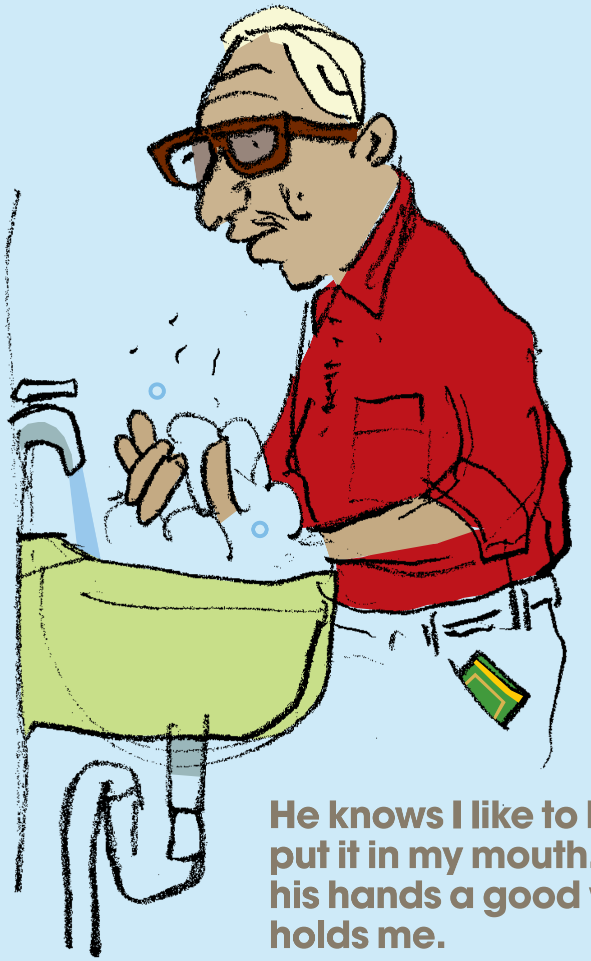

He knows I like to hold his finger and put it in my mouth. He always gives his hands a good wash before he holds me.

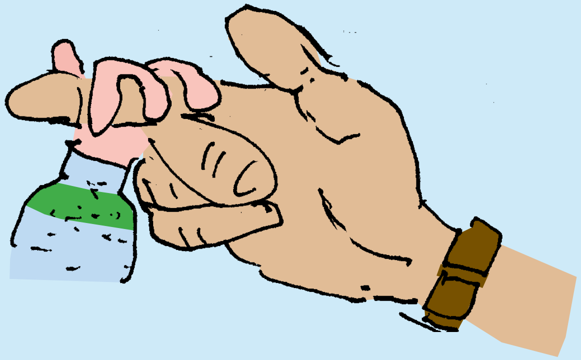

DEVICE 4 A5 .indd 10-11 2/4/12 10:39:29

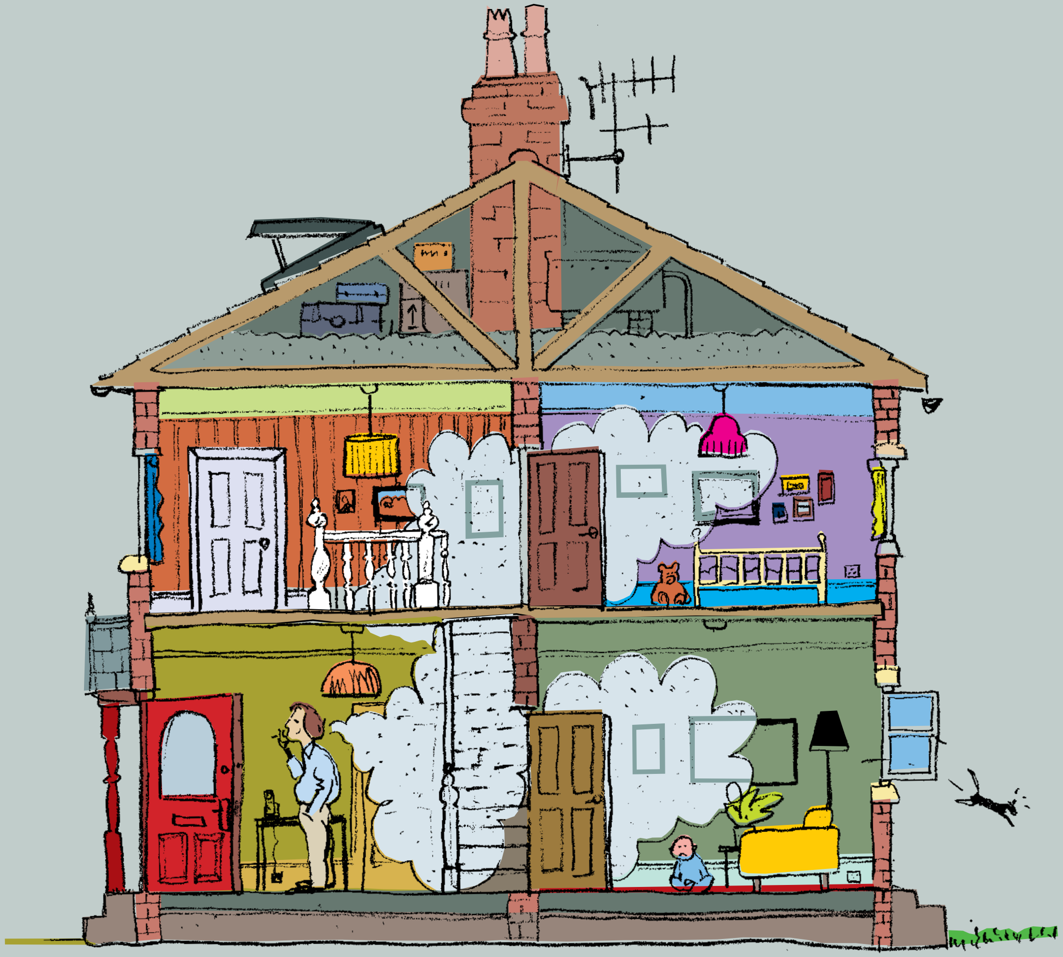

Please remember:
if you smoke in the
house I smoke too.

A big Thank You to all my
new friends and relatives
for protecting me from
Second Hand Smoke!

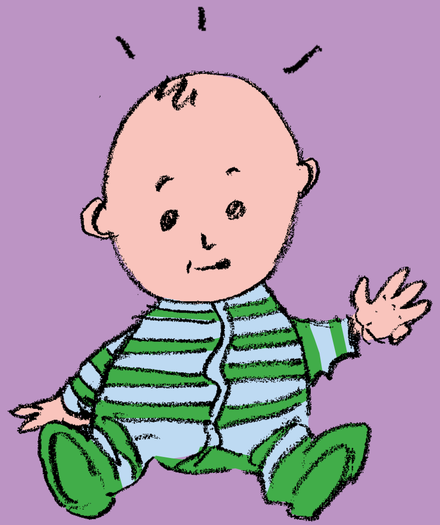

SECOND HAND SMOKE IS AN INVISIBLE THREAT

Second Hand Smoke is the smoke which comes from the burning end of a cigarette and the smoke blown out by smokers.

The smoke is full of chemicals and poisons, including arsenic, lead and cyanide.

Babies have no choice— if you smoke in the room, they smoke too.

Babies depend on you to protect them from Second Hand Smoke

Do you want to stop smoking? If you do, the NHS can provide support for you.

Call 0800 169 4219 for Leeds NHS Stop Smoking Service

Protect me... From Second Hand Smoke

NHS

DEVICE 4 A5 .indd 14 2/4/12 10:39:30
