# Supplementary material for: The challenge of mothers learning about secondhand smoke (MLASS): a quasi-experimental, mixed methods feasibility study
Source: Pilot Feasibility Stud. 2016 Feb 6;2:9. doi: 10.1186/s40814-016-0048-0 (PMC5153670; doi:10.1186/s40814-016-0048-0)
Supplement: Additional file 4: — Post hoc survey. (DOCX 23 kb) [file 40814_2016_48_MOESM4_ESM.docx]

Additional file 4

We would be grateful if you would take just a few moments to complete this short survey. You will NOT be identified from this survey. Please circle your answer to each question.

**Q1. At the present time, do you smoke?**

**YES NO**

**Q2. Do you currently live with someone who smokes and/or have visitors who smoke?**

**YES NO**

**IF YOU ANSWERED “YES” TO Q1 OR Q2”, THEN PLEASE ANSWER THE FOLLOWING QUESTIONS**

**Q3. Have you received any information about smoke free homes? (Please tick)**

**Yes I have received information from my midwife**

**Yes I have received information from another source (please specify)……………………………**

**No I have not received any information about smoke free homes**

**Q4. Are there any restrictions or limits on where smoking can take place in your house? (Please tick)**

**My home was smoke free even before I found out I was pregnant**

**My home has been smoke free since I found out I was pregnant**

**There are NO limits or restrictions on smoking in my house**

***Thank you for completing this survey***
