# Supplementary material for: The challenge of mothers learning about secondhand smoke (MLASS): a quasi-experimental, mixed methods feasibility study
Source: Pilot Feasibility Stud. 2016 Feb 6;2:9. doi: 10.1186/s40814-016-0048-0 (PMC5153670; doi:10.1186/s40814-016-0048-0)
Supplement: Additional file 5: — Adherence by health profesionals to intervention delivery. (DOCX 17 kb) [file 40814_2016_48_MOESM5_ESM.docx]

Additional file 5

| Participant & age at study entry (baseline) | Self-reported Smoking status | Community midwife delivered interventions | Heath visitor delivered interventions |
| --- | --- | --- | --- |
| 001  Age:18 | Smoker | A: not applicable  C: given @ antenatal appointment @40 weeks +3 days | B: given @ early start visit  D: given postnatal day 10 |
| 002  Age:20 | Non-smoker | A: not known  C: given postnatal day 13 | B: given @ early start visit  D: given postnatal day 11 |
| 003  Age:28 | Smoker | A: not applicable  C: Not given - participant did not attend postnatal day 3 appointment with midwife | B: posted with red book  D: given postnatal day 14 |
| 004  Age:17 | Smoker | A: not applicable  C: Not given – midwife did not visit until postnatal day 12 | B: given @ early start visit  D: given postnatal day 11 |
| 005  Age:21 | Non-smoker | A: given @16 week antenatal appointment  C: not given due to postal issues when GP surgery closed | B: posted with child health book by HV administration team  D: given postnatal day 12 |
| 006  Age:30 | Non-smoker | A: given @16 week antenatal appointment  C: given @ 39 week antenatal appointment. (planned Caesarean-section) | B: given @ early start visit  D: given postnatal day 13 |
| 007  Age: 22 | Non-smoker | A: given @16 week antenatal appointment  C: given on postnatal day 3 | B: given @ early start visit  D: given postnatal day 11 |
| 008  Age:18 | Smoker | A: not applicable  C: Not given | B: given at routine visit by the family nurse practitioner  D: given postnatal day 10 |
